# Recent Advances in Air-Stable *n*-Type Single-Walled Carbon Nanotube Composites for Thermoelectric Applications

**DOI:** 10.3390/ma19143065

**Published:** 2026-07-16

**Authors:** Asumi Eguchi, Kento Sunaga, Masayuki Takashiri

**Affiliations:** Course of Applied Chemistry, Graduate School of Engineering, Tokai University, Hiratsuka 259-1292, Kanagawa, Japan; 6cajm007@tokai.ac.jp (A.E.); sunaga.k.32fe@m.isct.ac.jp (K.S.)

**Keywords:** single-walled carbon nanotubes (SWCNTs), thermoelectric conversion, thermoelectric materials, *n*-type doping, *n*-type characteristics, air stability, atmospheric stability, composite materials, nanocomposites, flexible electronics, wearable devices

## Abstract

**Highlights:**

- Three composite strategies yield air-stable *n*-type SWCNTs with high thermoelectric power factors.
- Inorganic, polymer, surfactant, and bio-derived coatings physically block oxygen for long-term stability.
- Dynamic covalent networks and elastomers add flexibility for thousands of bending cycles.
- Conformal, flexible thermoelectric modules become feasible for wearables and IoT sensor networks.
- Low-impact aqueous and amino-acid processes support sustainable device manufacturing.

**Abstract:**

With the rapid advancement of the IoT society and growing awareness of environmental issues, thermoelectric conversion technology—which directly converts waste heat into electricity—is gaining attention as a self-powered, autonomous power source capable of driving countless devices. While currently mainstream metal-based inorganic thermoelectric materials demonstrate high performance, their high rigidity and brittleness, as well as their frequent inclusion of toxic heavy metals, have limited their application in biological systems and on curved surfaces. As a next-generation alternative, single-walled carbon nanotubes (SWCNTs)—which possess excellent flexibility, electrical conductivity, and mechanical strength while being low in toxicity—are garnering significant attention. However, *n*-type SWCNT materials, which are essential for thermoelectric module fabrication, have faced two major barriers to practical application: low atmospheric stability (they easily revert to *p*-type upon exposure to atmospheric oxygen and moisture) and thermoelectric performance that falls short of inorganic materials. This review comprehensively outlines the latest composite approaches designed to overcome these critical challenges and achieve both extreme atmospheric stability and high thermoelectric performance in *n*-type SWCNT materials, along with the flexibility required to withstand severe deformation. Three main strategies are discussed. The first is the organic/polymer approach, which involves doping with organic small molecules that control the LUMO level or bicyclic organic superbases with strong electron-donating properties, as well as polymer coating, to achieve long-term stable *n*-type characteristics and high power output even in air or under severe high-temperature conditions. The second is the inorganic hybrid strategy, which involves nanoscale compositing with inorganic materials such as Bi_2_Te_3_ and Cu_2_O; this reduces thermal conductivity through phonon scattering via interface control, while the inorganic layer physically blocks oxygen to ensure long-term atmospheric stability. The third approach involves ultra-long-term stabilization techniques, such as bulk encapsulation using cationic or gemini surfactants, and environmentally friendly aqueous processes utilizing natural amino acids. Furthermore, we discuss the latest developments in imparting practical-level toughness (flexibility) capable of withstanding thousands of bending cycles and high tensile stress through the introduction of dynamic covalent network polymers and elastomers. The conformal flexible thermoelectric power generation modules created through the integration of composite optimization, low-environmental-impact processes, and doping techniques will serve as a crucial foundational technology for realizing a sustainable next-generation electronics society, including future wearable devices, artificial skin, and smart sensor networks.

## 1. Introduction

### Background and Motivation

Environmental degradation and the depletion of fossil fuels have long been identified as serious global problems. The massive use of fossil fuels, coupled with mass production and consumption, has driven rising energy demand due to global population growth, economic expansion in emerging economies, and the recent surge in IoT (Internet of Things) devices. The combustion of fossil fuels releases large amounts of greenhouse gases, such as carbon dioxide (CO_2_), and waste heat, contributing to global warming and climate change. Furthermore, the increase in industrial activities, household waste, plastics, and chemicals is leading to the progressive pollution of air, water, and soil, raising concerns about biodiversity loss and adverse health effects [1].

The proliferation of IoT devices presents additional challenges: constructing and maintaining power grids to drive countless sensors, along with the enormous costs associated with the regular replacement and disposal of primary batteries, are problems that must be addressed immediately. Consequently, there is growing demand for compact, self-powered devices that can convert unused ambient energy into electricity autonomously. In response, the United Nations has established the Sustainable Development Goals (SDGs) and is promoting solutions across the international community. The background described above is deeply related to SDG 7 (affordable clean energy), SDG 9 (sustainable industrialization and innovation), SDG 12 (sustainable consumption and production), and SDG 13 (climate action) [2,3,4].

Thermoelectric conversion technology can directly convert not only high-temperature waste heat from factories and automobiles but also low-grade waste heat—such as the slight temperature differences found in body heat—into electricity. Because it contains no mechanical moving parts, it is exceptionally quiet and maintenance-free. The system can also be easily miniaturized and made lighter. Consequently, there are high expectations for its use as an autonomous power source for IoT devices.

The performance of thermoelectric conversion materials is quantified by the dimensionless figure of merit (*ZT*), expressed as:

(1) $$ZT=\frac{S^{2}\sigma }{\kappa }T$$

In Equation (1), *S* represents the Seebeck coefficient, *σ* represents electrical conductivity, *κ* represents thermal conductivity, and *T* represents absolute temperature. The product *S*^2^*σ* is referred to as the power factor (*P.F.*), which serves as a guideline for output. As is evident from Equation (1), thermoelectric conversion materials require both high electrical conductivity and a high Seebeck coefficient, as well as low thermal conductivity. However, materials with high electrical conductivity also tend to have high thermal conductivity because carriers transport heat; moreover, increasing carrier concentration improves electrical conductivity but reduces the Seebeck coefficient. Due to these inherent trade-offs, independently controlling these parameters is extremely difficult—a central challenge in thermoelectric materials research for decades [5,6].

Metal-based inorganic materials, which still account for the majority of thermoelectric devices in practical use today, have achieved high performance through decades of sustained research and development, as illustrated by the following representative examples.

Among *p*-type materials, polycrystalline tin selenide (SnSe) has set a new record for bulk thermoelectric materials through an impurity-removal process that thoroughly eliminates surface tin oxide (SnO_x_); this purification reduced the lattice thermal conductivity to an ultra-low value of approximately 0.07 W/(m·K) at 783 K, below even that of single crystals. Doping with 3 at% Na further improved the electrical conductivity (*σ*) while optimizing the Seebeck coefficient (*S*; maximum +342 μV/K), together yielding a maximum *ZT* of approximately 3.1 at 783 K and a power factor of ~12.06 μW/(m·K^2^) at 473 K—the highest value yet reported for polycrystalline SnSe [7].

A similarly high level of performance has been reported for Bi-Sb-Te (BST) materials: spark-plasma-sintered (SPS) samples reached an in-plane power factor of approximately 3540 μW/(m·K^2^) near room temperature and a maximum *ZT* of 1.2 at 373 K, making BST a standard high-performance material in the low-to-medium temperature range [8].

In *n*-type Mg_3_(Sb,Bi)_2_, incorporating metal nanoinclusions such as niobium (Nb) and tantalum (Ta) reduces interfacial barriers at grain boundaries, improving *σ* near room temperature; this approach yields a power factor exceeding 30 μW/(m·K^2^) at room temperature, a maximum *ZT* of 2.04 at 798 K and an average *ZT* of 1.57 over 300–798 K [9].

Likewise, for *n*-type PbTe, a band-flattening strategy based on manganese (Mn) doping increases the effective mass and improves the *S* value (maximum −92.1 μV/K at 323 K); a 0.5 at% Mn-doped sample achieved a power factor of 20.8 μW/(m·K^2^) at 573 K and a maximum *ZT* of 1.0 at 773 K [10].

Despite these impressive achievements, most metal-based inorganic thermoelectric materials remain highly rigid and brittle, complicating their processing, and many contain toxic heavy metals such as lead and tellurium that restrict their use in applications involving human contact. These limitations continue to drive demand for flexible, non-toxic thermoelectric materials.

In response, single-walled carbon nanotubes (SWCNTs)—carbon-based materials composed solely of carbon atoms—have recently attracted considerable attention. SWCNTs possess high electrical conductivity and flexibility and are easy to process. They also have the mechanical strength necessary for practical applications such as conforming to curved surfaces (a pure SWCNT film alone has a tensile strength of approximately 16.5 MPa). Furthermore, as will be discussed later, this strength can be significantly improved by compounding them with polymers, etc., making them a promising candidate for next-generation thermoelectric materials [11,12,13].

For thermoelectric module fabrication, connecting *p*-type and *n*-type elements alternately in series is essential. Regarding *p*-type SWCNT materials, stability has been achieved not only through natural *p*-type doping caused by the adsorption of atmospheric oxygen but also through doping effects. For example, ‘soft anions’ such as TFSI^−^ and NFSI^−^ have maintained *p*-type characteristics for over one year (more than 9000 h) in air at 373 K, demonstrating durability even at elevated temperatures [14].

On the other hand, *n*-type SWCNT materials continue to face two major challenges: long-term atmospheric instability (easy reversion to *p*-type due to oxygen and moisture exposure) and thermoelectric performance that remains below that of inorganic materials. Ongoing research and development aim to address these limitations, and this review comprehensively describes the strategies that have been established in recent years.

## 2. Mechanisms for Improving the Thermoelectric Properties of *n*-Type SWCNT Composites

This section summarizes the mechanisms underlying the improvement of thermoelectric properties in *n*-type SWCNT composites, organized into three groups corresponding to the composite strategies discussed in Section 4.

### 2.1. Organic/Polymer Composites

SWCNTs inherently exhibit *p*-type characteristics due to the adsorption of atmospheric oxygen. In organic/polymer composites, the lone electron pairs of nitrogen (N) and oxygen (O) atoms present in organic small molecules and polymers function as strong electron donors. These lone pairs fill the holes in the valence band of SWCNTs and promote electron injection into the conduction band, thereby converting the primary carriers from holes to electrons. This results in the manifestation of *n*-type thermoelectric properties through an upward shift in the Fermi level [15,16,17].

At the interface between SWCNTs and organic materials, precisely controlling the energy levels (HOMO and LUMO) of both materials can create an energy barrier. This energy-filtering effect selectively scatters low-energy carriers while allowing high-energy carriers to pass through, thereby improving the Seebeck coefficient [18,19,20].

Furthermore, a structure in which polymer chains densely coat (wrap) the SWCNT surface acts as a physical barrier that suppresses the adsorption of atmospheric oxygen molecules, thereby dramatically improving the long-term atmospheric stability of *n*-type characteristics [15,21,22].

Organic small molecules and π-conjugated polymers with planar structures form strong π–π interactions or cation–π interactions with SWCNTs, effectively suppressing SWCNT aggregation and maintaining a uniform dispersion. This enables the formation of a continuous charge transport network, contributing to high electrical conductivity [18,23]. In non-conjugated polymer systems, hydrogen bonds are thought to form between oxygen atoms in the polymer backbone and the SWCNTs, lowering the energy barrier for electron tunneling and promoting efficient electron transfer pathways [24,25,26].

In summary, compositing organic materials with SWCNTs yields four key effects: (1) N and O atoms donate electrons to SWCNTs, converting carriers from holes to electrons and inducing *n*-type behavior; (2) the interfacial energy barrier filters out low-energy carriers, improving the Seebeck coefficient; (3) the polymer coating prevents oxygen adsorption, enabling long-term *n*-type stability in air; (4) π–π interactions and related forces uniformly disperse SWCNTs, forming a network that improves electrical conductivity.

### 2.2. Inorganic Hybrid Composites

Representative inorganic hybrid systems combine SWCNTs with inorganic semiconductors such as bismuth telluride (Bi_2_Te_3_), tin selenide (SnSe), and copper oxide (Cu_2_O).

Using approaches such as covalent grafting via solvothermal synthesis, Sn–C covalent bonds can be directly formed between inorganic atoms and the carbon atoms of SWCNTs. In this method, inorganic atoms bond with the SWCNTs in place of oxygen atoms, thereby eliminating oxygen interference; electrons are drawn toward the SWCNT side, leading to robust and stable *n*-type behavior [27].

By combining inorganic semiconductors—which inherently possess a high *n*-type Seebeck coefficient—with SWCNTs, which exhibit high electrical conductivity, the complementary properties of both materials can be leveraged synergistically, resulting in significant improvement in power factor [28,29].

Furthermore, the discontinuous chemical bonds and numerous interfaces formed at the interface between SWCNTs and inorganic nanocrystals (such as nanoplates and microspheres), as well as the defects introduced during compositing, strongly scatter phonons (lattice vibrations) responsible for heat transport. This phonon scattering dramatically reduces the thermal conductivity—a major weakness of SWCNTs—while maintaining electrical conductivity, contributing significantly to improved thermoelectric conversion efficiency [27,30,31].

In summary, compositing inorganic semiconductors with SWCNTs provides three key benefits: (1) direct Sn–C (or equivalent) covalent bond formation via solvothermal synthesis supplies electrons to SWCNTs while eliminating oxygen interference, achieving robust *n*-type characteristics; (2) the high *n*-type Seebeck coefficient of the inorganic semiconductor combined with the high electrical conductivity of SWCNTs synergistically improves the power factor; (3) interfacial defects and discontinuous chemical bonds strongly scatter phonons, dramatically suppressing the high thermal conductivity without compromising electrical conductivity.

### 2.3. Surfactant and Bio-Derived Composites

Surfactants not only improve the dispersibility of SWCNTs but also act as dopants when specific ions are present, and they serve to protect SWCNTs from atmospheric oxygen.

In systems using cationic surfactants (such as CTAB, TBAB, and DODMAC), the halide counterions (Cl^−^, Br^−^, I^−^, etc.) act as strong electron donors, causing electron injection into the SWCNTs. For example, during processes such as ultrasonic irradiation, Br^−^ has been confirmed to donate electrons to SWCNTs, forming Br_2_ in the process, resulting in *n*-type doping [32,33,34].

In systems using anionic surfactants such as SDBS, after adsorbed oxygen is desorbed by heat treatment, sodium ions (Na^+^) contained in the hydrophilic groups of the surfactant are attracted to the SWCNT surface in high density due to electrostatic forces. Electron transfer from these Na atoms to the SWCNTs is believed to promote the manifestation of *n*-type characteristics [34].

By forming a high-density, uniform adsorption layer on the SWCNT surface, surfactant molecules function as a physical barrier (bulk encapsulation) that prevents oxygen intrusion, thereby achieving long-term atmospheric stability lasting from several months to years. This stability depends on the size of the counterion in cationic surfactants: smaller counterions (e.g., Cl^−^ rather than I^−^) form a denser and more robust protective layer. Furthermore, gemini surfactants (such as 12-3-12), which possess two hydrophobic groups and one hydrophilic group, exhibit significantly higher adsorption capacity toward SWCNTs compared to conventional single-chain surfactants, blocking oxygen access more effectively by densely coating the surface [35,36,37].

When crown ethers (such as DB18C6) capture alkali metal ions (such as K^+^) to form complexes ([K(DB18C6)]^+^), a ‘naked anion’ with high reducing power is generated. This anion efficiently donates electrons to the carbon framework of the SWCNT, significantly shifting the Fermi level toward the conduction band and achieving *n*-type conversion [38,39,40].

In bio-derived systems, natural basic amino acids such as lysine function as environmentally friendly *n*-type dopants due to their polar amino groups, promoting electron injection into SWCNTs. Furthermore, the polar functional groups of amino acids (amino and carboxyl groups) form a robust hydrogen-bonding network with elastomer main chains such as aqueous polyurethane. This network effectively suppresses relative displacement and structural damage between SWCNTs, enabling the composite material to maintain high thermoelectric performance while imparting excellent stretchability (maximum strain tolerance: ~29%)—essential for wearable device applications [25].

## 3. Strategy for Composite Materials Using *n*-Type SWCNTs

As described in the Introduction, thermoelectric power generation is attracting attention as a technology that directly converts unused ambient or body heat into electricity. Due to their excellent electrical conductivity, mechanical flexibility, light weight, and low toxicity, SWCNTs are considered the leading candidate for next-generation flexible thermoelectric materials. However, SWCNTs typically exhibit strong *p*-type characteristics because electrons are lost due to the adsorption of atmospheric oxygen; even after conversion to *n*-type using strong electron donors (dopants), they often revert to *p*-type due to oxygen and moisture in the atmosphere. Consequently, maintaining high-performance *n*-type SWCNTs stably over long periods under atmospheric conditions has been the greatest challenge in this field for many years [13,41,42,43,44,45].

A highly effective approach to overcoming this challenge while simultaneously addressing the intrinsic weaknesses of pure SWCNTs—their high thermal conductivity and low Seebeck coefficient—is compositing them with other materials and employing precise molecular doping strategies. Composite formation not only enables *n*-type conversion through efficient electron injection but also promises synergistic effects: improved Seebeck coefficients via energy-filtering effects at interfaces between dissimilar materials, and reduced thermal conductivity due to phonon scattering. Furthermore, densely coating (encapsulating) the SWCNT surface with composite polymers or molecules has been shown to physically and chemically block oxygen intrusion, leading to significant long-term stabilization of *n*-type characteristics [21,30,35,46,47].

In this section, we focus on three main approaches for realizing high-performance and highly stable *n*-type SWCNT flexible thermoelectric materials: (1) organic, polymer, and polymer-based systems; (2) inorganic systems; (3) special composite, surfactant, and bio-derived systems. We outline the improvement mechanisms and the latest material design strategies for each approach.

### 3.1. Inorganic-Metal Nanomaterials and Hybrids

Research on inorganic hybrid composite materials has evolved with the aim of synergistically combining the high Seebeck coefficient of inorganic materials with the flexibility and low thermal conductivity of carbon-based materials [45,46].

Early polymer–inorganic composite approaches attempted to fill arrays of vertical silicon nanowires with insulating polymers, aiming to increase the Seebeck coefficient while reducing thermal conductivity. Although this method succeeded in lowering thermal conductivity, it caused a significant decrease in electrical conductivity—a major drawback [48].

To prevent this decrease in electrical conductivity, the research focus shifted from insulating polymers to conductive polymers such as PEDOT:PSS, as well as to SWCNTs combined with inorganic materials such as Bi_2_Te_3_ and Sb_2_Te_3_ [48,49,50]. However, simply mixing materials in solution produced extremely high contact resistance at the interface between inorganic nanomaterials and CNTs, leading to decreased overall conductivity of the composite. To resolve this issue and dramatically improve thermoelectric performance, researchers developed advanced fabrication processes that integrate materials at the nanoscale—going beyond simple mixing [51].

The following three approaches represent the latest design strategies for realizing high-performance and stable *n*-type SWCNT composites.

**Figure 1:**
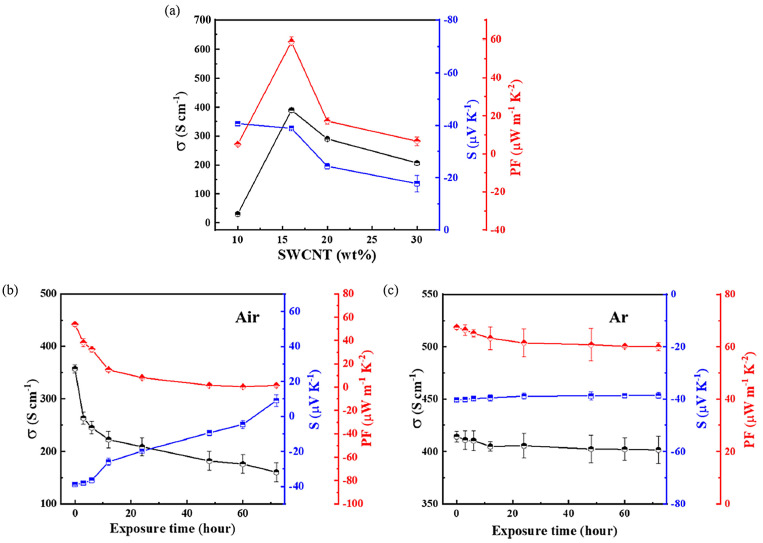
(**a**) Thermoelectric properties of *n*-type SWCNT/SnSe nanocomposite films as a function of SWCNT weight fraction at room temperature. (**b**) Stability of thermoelectric properties in air. (**c**) Stability of thermoelectric properties in an Ar atmosphere. Reprinted with permission from Ref. [27]. Copyright 2021 American Chemical Society.

Approach 1: Covalent grafting via the solvothermal method. Fan et al. reported on an *n*-type flexible thermoelectric film in which SnSe nanocrystals were grafted onto SWCNTs (single-walled carbon nanotubes) using a simple solvothermal method. The specific fabrication process involved first heating a mixed solution containing SWCNTs, tin and selenium precursors, a reducing agent (hydrazine monohydrate), and a surfactant (PVP) in an autoclave at 453 K for 12 h (solvothermal reaction). Subsequently, the resulting product was vacuum-filtered while being washed with ethanol and pure water, then dried to produce a self-supporting flexible film. In this in situ synthesis process, Sn-C covalent bonds were formed directly between SWCNTs and SnSe, thereby eliminating the influence of oxygen and achieving *n*-type characteristics. As shown in Figure 1a, at an optimized SWCNT content of 16 wt% in the composite material, a maximum power factor of 58.86 [μW/(m·K^2^)] was achieved at room temperature. Furthermore, as shown in Figure 1b,c, although a decline in performance was observed after approximately 70 h of long-term storage in air—resulting in a transition to *p*-type—the material exhibited exceptional long-term stability under an argon atmosphere, with thermoelectric performance remaining constant and showing almost no dependence on exposure time even after more than 70 h [27].

**Figure 2:**
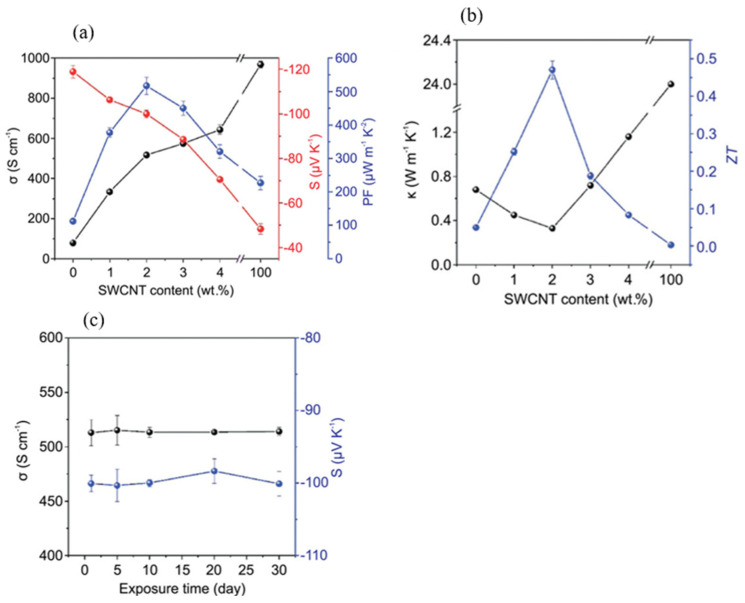
(**a**) Electrical conductivity, Seebeck coefficient, and *P.F*. of the Bi_2_Te_3_/SWCNT (2 wt%) hybrid film at 300 K. (**b**) Effect of SWCNT content on in-plane thermal conductivity and *ZT*. (**c**) Stability of electrical conductivity and Seebeck coefficient in air. Reprinted from Ref. [52] under the CC BY license.

Approach 2: Fabrication of a “cement-rebar” structure via a solution process. Chen et al. developed *n*-type Bi_2_Te_3_/SWCNT hybrid films using a solution process amenable to mass production. Bi_2_Te_3_ nanoplates were composited with ethylenediamine-treated SWCNTs at an optimal SWCNT content of 2 wt%. A precursor ionic solution of Bi_2_Te_3_ dissolved in a mixed solvent of ethylenediamine (en) and 1,2-ethanedithiol (edt) was mixed with a SWCNT dispersion, cast, and dried. The resulting film was then immersed in ethylene glycol (EG) and annealed at 653 K. As shown in Figure 2a,b, at 300 K, the material exhibited an electrical conductivity of 517.2 S/cm, a Seebeck coefficient of −100 μV/K, a power factor of 517.2 μW/(m·K^2^), and a thermal conductivity of 0.33 W/(m·K), achieving a *ZT* value of 0.47. As shown in Figure 2c, almost no decay in electrical conductivity or Seebeck coefficient was observed after 30 days of air exposure, demonstrating excellent air stability [52].

**Figure 3:**
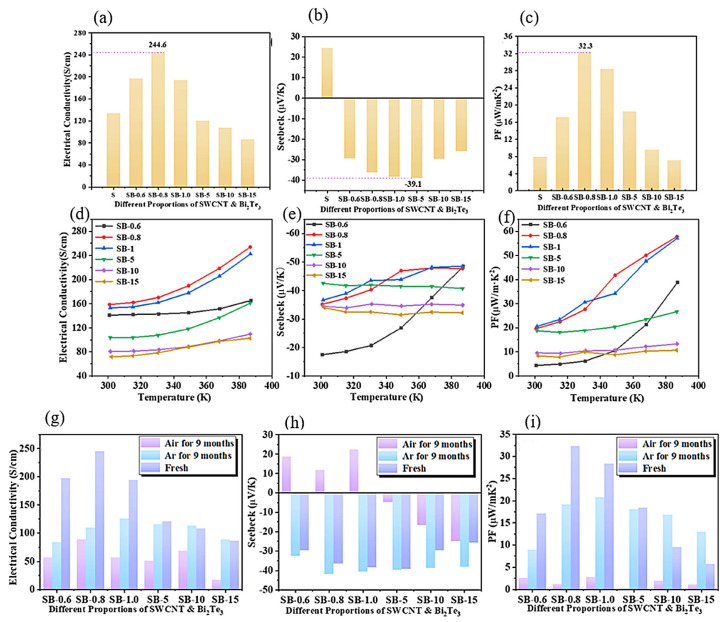
(**a**–**f**) Thermoelectric properties of SWCNT composite films with varied Bi_2_Te_3_ loading and temperature dependence. (**g**–**i**) Electrical conductivity, Seebeck coefficient, and *P.F*. after 9 months in air or argon. Reprinted with permission from Ref. [53]. Copyright 2021 American Chemical Society.

Approach 3: In situ solvothermal synthesis assisted by PVP. Chen et al. proposed a method for directly growing Bi_2_Te_3_ nanosheets on a SWCNT network using PVP as a stabilizer. The optimal mass ratio was SWCNT:Bi_2_Te_3_ = 1:0.8 (designated SB-0.8), with ethylene glycol (EG) as the solvent. SWCNTs were dispersed in EG containing PVP; precursor salts of Bi and Te were added; and a solvothermal reaction was performed at 453 K for 12 h, followed by vacuum filtration to yield a composite film. As shown in Figure 3a–f, the composite exhibited excellent *n*-type thermoelectric performance at 386 K: electrical conductivity of 253.9 S/cm and a power factor of 57.8 μW/(m·K^2^). Regarding long-term stability, as shown in Figure 3g–i, the decrease in electrical conductivity was less than 13% after 100 h of storage in air, and no decrease in the Seebeck coefficient was observed. However, performance degradation due to oxidation was confirmed after 9 months of storage in air, whereas no degradation occurred after 9 months of storage in an argon atmosphere [53].

The strategy of firmly compositing or coating SWCNTs with inorganic materials such as Bi_2_Te_3_ and Cu_2_O offers a groundbreaking solution to oxidative degradation in air—the greatest weakness of *n*-type SWCNT thermoelectric materials. Both solvent-based wet processes and dry processes combined with plasma treatment now allow inorganic crystals to be grown in situ while preserving the conductive pathways of the SWCNT network. Materials with a surface-protection structure, in which the inorganic layer physically blocks contact with oxygen, have effectively prevented re-*p*-type conversion even after long-term air exposure ranging from 30 to 210 days. This inorganic hybrid strategy is therefore expected to develop further as a key approach for the practical application of wearable thermoelectric devices that combine high performance with excellent air stability and flexibility.

Other approaches utilizing inorganic metal hybrids are summarized in Table 1.

### 3.2. Special Coatings, Biomolecules, and Surfactants

To overcome the challenge of *n*-type atmospheric instability, approaches utilizing biomolecules, special coatings, and surfactants have each evolved into active areas of research.

The use of biomolecules began with early attempts to insert biomolecules into the network junctions of SWCNTs. In recent years, this has evolved into environmentally friendly processes utilizing natural amino acids such as lysine. By combining these with elastic polymers, highly biocompatible materials have been developed that maintain high thermoelectric performance while achieving the excellent stretchability and flexibility essential for wearable devices [25].

Surfactants were initially used solely as SWCNT dispersants; their role changed significantly after it was discovered that cationic surfactants themselves function as *n*-type dopants. Subsequent research has elucidated that surfactants act as a protective barrier by uniformly coating the SWCNT surface and preventing oxygen adsorption. Compounds such as dimethyldodecyl ammonium chloride (DODMAC) have achieved extremely long-term atmospheric stability lasting years by simultaneously serving as dopants and forming a robust physical barrier [35,36].

Increasingly, these approaches are being combined: uniform SWCNT dispersion and doping with surfactants, followed by specialized coating, together with the use of bio-derived molecules as dopants, are driving significant progress toward highly stable, high-performance, flexible wearable thermoelectric materials with environmentally friendly, simple manufacturing processes [35,39,58].

The following four representative studies illustrate these advances.

**Figure 4:**
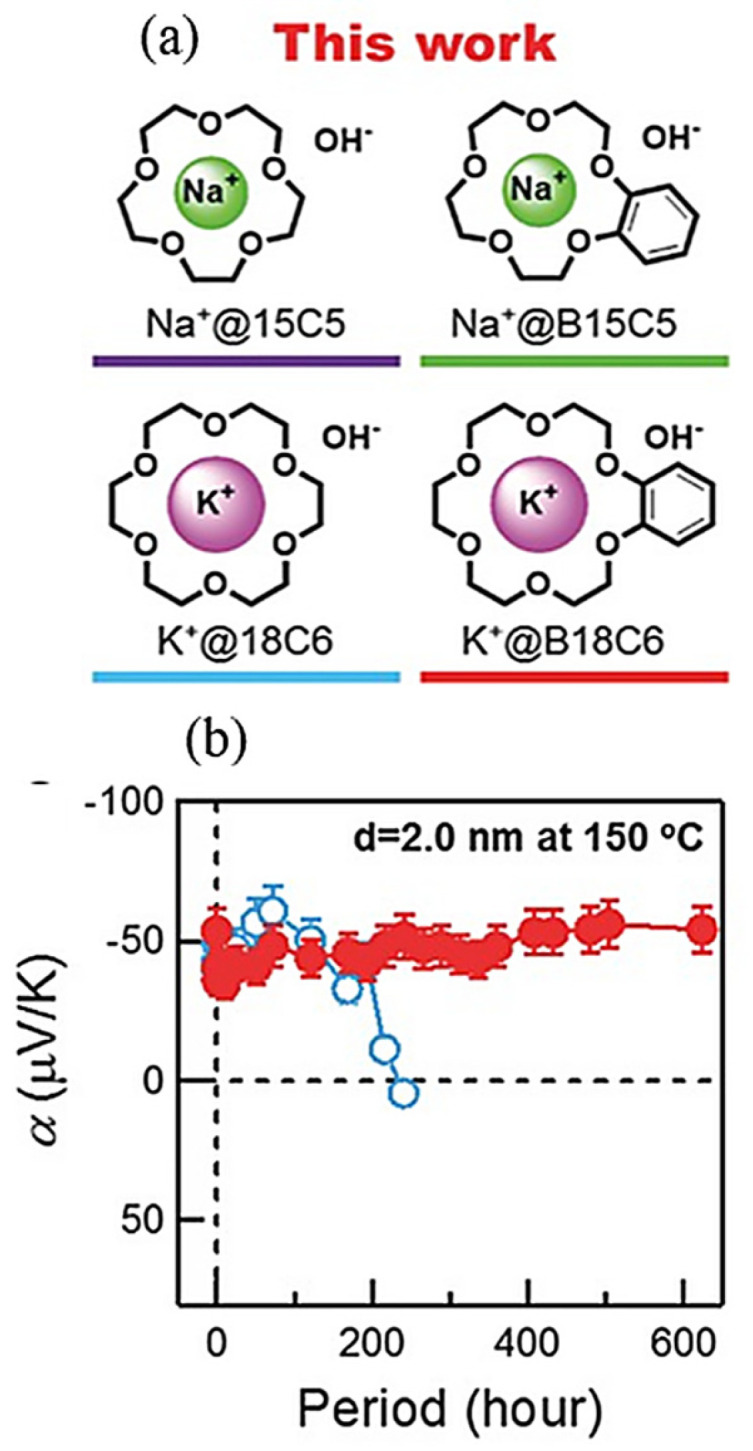
(**a**) Structural formula of the crown ether complex; (**b**) Changes in Seebeck coefficient over time at 423 K. Reprinted with permission from Ref. [58]. Copyright 2016 Wiley-VCH Verlag GmbH & Co. KGaA, Weinheim.

Method 1: Highly stable doping using alkali salt and crown ether complexes. Nonoguchi et al. (2016) [58] discovered that simple complexes of bases and crown ethers function as stable *n*-type dopants for CNTs. Using potassium hydroxide (KOH) and benzo-18-crown-6-ether (or onium salts such as TMAOH) in methanol or DMF, a self-supporting CNT film (bucky paper) was prepared by vacuum filtration of an ultrasonically treated CNT dispersion and then doped by immersion in a 0.1 mol/L mixed solution of salt and crown ether for ~10 min, followed by washing and drying. The structural formula is shown in Figure 4a. When eDIPS-SWCNT was treated with KOH/18-crown-6-ether, the in-plane power factor reached 230 μW/(m·K^2^) and the conductivity reached 2.05 × 10^3^ S/cm; the dimensionless through-plane transport coefficient reached *ZT* = 0.1 at 423 K or higher. In terms of long-term stability, as shown in Figure 4b, the system using benzo-18-crown-6-ether and KOH remained stable for more than 700 h (~1 month) in air at 373 K, and maintained *n*-type characteristics for more than 600 h at 423 K [58].

**Figure 5:**
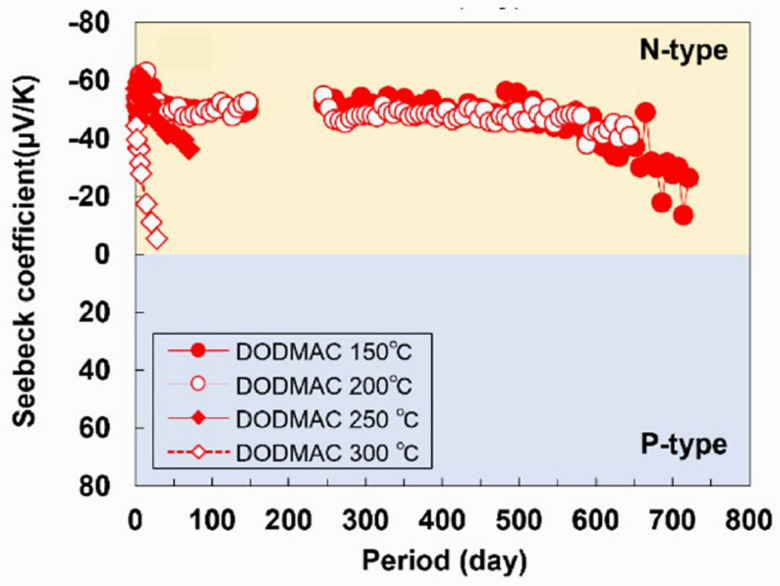
Time-dependent changes in the Seebeck coefficient of DODMAC/CNT films heat-treated at different temperatures. Reprinted from Ref. [36] under the CC BY license.

Method 2: Ultra-long-term atmospheric stabilization using cationic surfactants. Amma et al. (2022) [36] reported an all-carbon thermoelectric module with record-breaking ultra-long-term atmospheric stability by utilizing the hydrophobic interactions of cationic surfactants. They used 0.2 wt% SWCNT (ZEONANO SG101) and 1.0 wt% of DODMAC or CPC as cationic surfactants in deionized water. The dispersion was drop-cast onto a glass substrate (2.5 × 2.0 cm), air-dried for ~24 h, and then heat-treated at the optimal temperature (423 K for DODMAC) for 1 h. The DODMAC/SWCNT film heat-treated at 423 K exhibited a power factor of 3.6 μW/(m·K^2^) at 300 K, with an extremely low in-plane thermal conductivity of 0.62 W/(m·K) and a *ZT* of 1.7 × 10^−3^. Notably, as shown in Figure 5, the device maintained a Seebeck coefficient of approximately −50 μV/K when left in ambient air at room temperature for 665 days and retained its *n*-type characteristics even after 721 days—among the longest atmospheric stabilities ever recorded [36].

**Figure 6:**
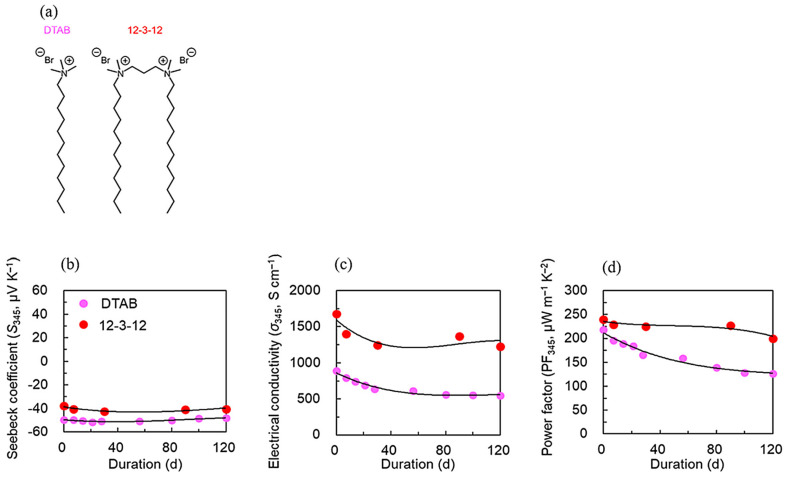
(**a**) Structural formulas of DTAB and 12-3-12. (**b**) Changes in the Seebeck coefficient over 120 days. (**c**) Changes in electrical conductivity over 120 days. (**d**) Changes in the power factor over 120 days. Reprinted with permission from Ref. [37]. Copyright 2022 American Chemical Society.

Method 3: Simultaneous achievement of high output and stability through gemini surfactant wrapping. Hata et al. (2022) [37] reported an aqueous process using gemini surfactants that further advances the coating structure of cationic surfactants. They used 60 mg of SWCNT (Meijo Nano Carbon) and 6.4 mM of gemini-type surfactants (12-3-12) and a single-chain surfactant (DTAB) for comparison in 30 mL of water. The structural formula is shown in Figure 6a. The mixture was dispersed for 15 min using an ultrasonic homogenizer in an ice bath, then filtered through a PTFE membrane filter to form a film. After washing four times with 1 L of water to remove excess surfactant, the membrane was air-dried for 3 h and then dried overnight in a vacuum oven (333 K, <0.1 MPa). The optimized 12-3-12/SWCNT film exhibited a Seebeck coefficient of −37.8 μV/K and a conductivity of 1678 S/cm, achieving a high power factor of 240 μW/(m·K^2^) (at 345 K) and a *ZT* of 6.04 × 10^−3^. As shown in Figure 6b, in air at 303 K and 65% relative humidity, the 12-3-12/CNT membrane retained 83% of its initial power factor after 120 days [37].

**Figure 7:**
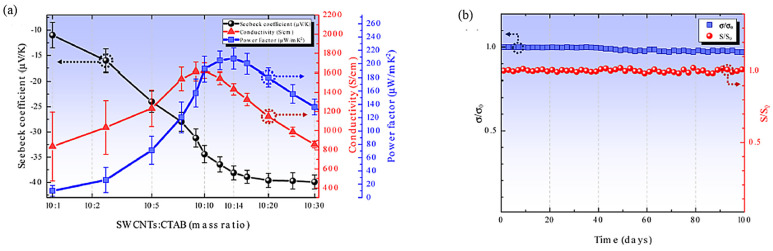
(**a**) Electrical conductivity, Seebeck coefficient, and *P.F*. at room temperature; (**b**) Long-term stability of *n*-type SWCNTs:CTAB (10:14 mg/mg) films in air without encapsulation. Reprinted with permission from Ref. [43]. Copyright 2021 American Chemical Society.

Method 4: Fully printed all-carbon thermoelectric generator via an aqueous process using cationic surfactants. Mytafides et al. reported a highly efficient, fully printed, all-carbon organic thermoelectric generator without metal junctions, fabricated using an environmentally friendly aqueous process. They used unmodified single-walled carbon nanotubes (SWCNTs) as the material, deionized water as the solvent, and CTAB, a cationic surfactant, as the *n*-type dopant. By mixing SWCNTs and CTAB at an optimal mass ratio of 10:14 (mg/mg) and subjecting the mixture to chip-type ultrasonic treatment, the researchers prepared a high-quality aqueous ink with a viscosity suitable for printing (approximately 350 cP). The device was fabricated by blade-coating this ink onto a flexible Kapton substrate and drying it at 363 K for 20 min. As a result, the CTAB-doped *n*-type SWCNT film exhibited a high power factor (*P.F.*) of 127 [μW/(m·K^2^)] at room temperature, as shown in Figure 7a, and reached 258 [μW/(m·K^2^)] at a temperature difference (Δ*T*) of 150 K (high-temperature side *T_h_* = 430 K). Regarding stability, as shown in Figure 7b, the film exhibited excellent long-term atmospheric stability even without encapsulation, with changes in thermoelectric properties over 100 days remaining below 3% [43].

The four methods discussed above represent groundbreaking approaches for overcoming the fatal oxidative degradation of *n*-type CNTs in air. Methods using salt/crown ether complexes or ionic liquids derived from organic superbases excel in strong interactions and high-temperature stability (long-term maintenance at 373–423 K), thereby achieving high power factors. Methods using cationic or gemini surfactants demonstrate significant strengths in their low environmental impact (aqueous process) and ultra-long-term room-temperature atmospheric stability achieved through physical oxygen blocking. Selecting the appropriate composite optimization, solvent process, and doping procedure according to the application will be key to the future development of practical flexible thermoelectric modules.

Additional approaches utilizing biomolecules, special coatings, and surfactants are summarized in Table 2.

### 3.3. Macromolecules, Polymers, and Organic Compounds

The development of thermoelectric materials produced by compositing SWCNTs with polymer-based and organic-based materials (such as organic small molecules) began with performance improvements in *p*-type materials, progressed through the major hurdle of *n*-type atmospheric stability, and has since evolved significantly into the flexible materials now used in wearable devices.

Research into the thermoelectric properties of polymers alone began in the 1970s. From the 1980s through the 1990s, extensive research was conducted on conductive polymers such as polyaniline (PANI); however, thermoelectric performance at that time remained low. Because the performance gap relative to conventional inorganic thermoelectric materials could not be ignored, hybrid materials combining organic and inorganic components emerged as a new research direction [72,73].

Following the discovery of carbon nanotubes in 1991, development progressed rapidly owing to their unique mechanical and electrical properties and excellent electron transport capabilities. In 1994, Ajayan et al. reported the first polymer nanocomposite reinforced with carbon nanotubes [74]. In the thermoelectric field, the integration of nanomaterials gained momentum in 2006, when Gupta and Miura synthesized PANI on SWCNTs via electrochemical polymerization and Meng et al. reported the first thermoelectric nanocomposite using PANI and multi-walled carbon nanotubes [73,74,75].

In recent years, research on thermoelectric polymers and their composites has advanced considerably. Historically, the organic components of flexible thermoelectric materials have primarily consisted of conductive polymers (CPs) such as PEDOT, PANI, P3HT, and PPy, and significant progress has been made in these *p*-type organic thermoelectric materials. By contrast, research on thermoelectric composites based on insulating thermoplastics currently lags far behind that on conductive-polymer-based materials. Nevertheless, organic–inorganic thermoelectric nanocomposites continue to attract significant attention because they integrate the high electrical conductivity of carbon materials (such as CNTs and graphene) with the low thermal conductivity and mechanical flexibility of polymers [25,31,72,75,76].

In polymer systems, electron donation from localized lone pairs results in *n*-type doping; amine and amide groups, which possess lone pairs on their nitrogen atoms, act as the electron donors.

The following four representative studies excel in both thermoelectric performance and long-term stability among SWCNT materials combining polymer-based and organic-based components.

**Figure 8:**
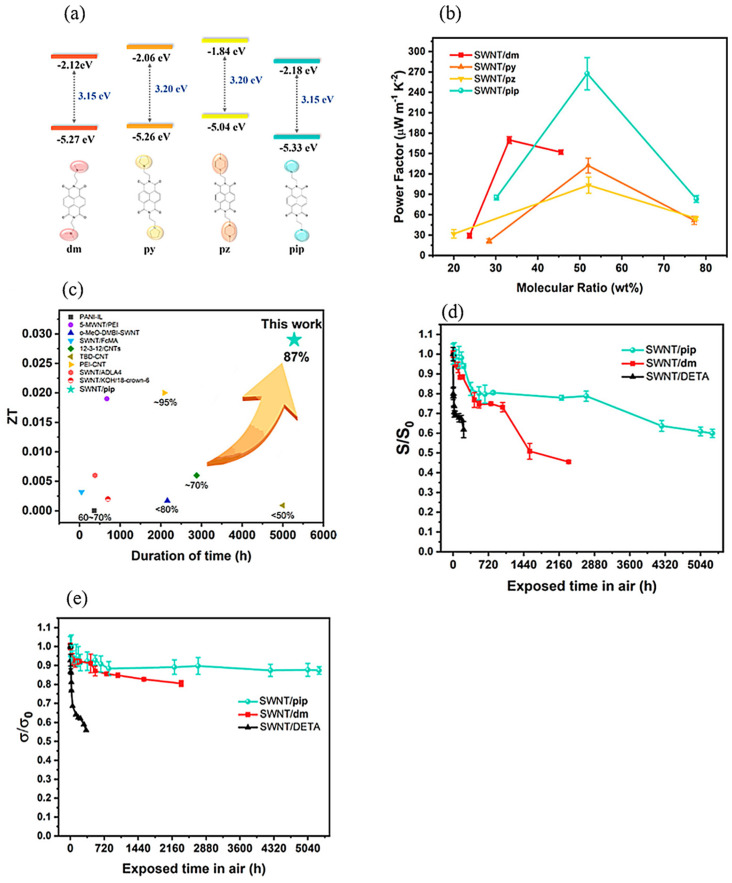
(**a**) Structural formula and energy levels of pip. (**b**) Power factors of untreated SWCNT, SWCNT/DETA, SWCNT/dm, SWCNT/py, SWCNT/pz, and SWCNT/pip. (**c**) Comparison of in-plane *ZT* values for various *n*-type organic and hybrid materials. (**d**) Time evolution of the *n*-type Seebeck coefficient ratio (*S*/*S*_0_). (**e**) Time evolution of the electrical conductivity ratio (*σ*/*σ*_0_). Reprinted with permission from Ref. [77]. Copyright 2023 American Chemical Society.

Method 1: Atmospheric stability through energy-level control of organic small molecules. Kim et al. (2023) [77] developed a highly air-stable *n*-type SWCNT hybrid material using organic small molecules (OSMs) with deeply controlled lowest unoccupied molecular orbitals (LUMOs). They used an OSM (designated pip), in which N-ethylpiperidinyl groups were introduced at both ends of a naphthalene diimide backbone, as a composite material for SWCNTs. The structural formula is shown in Figure 8a. The optimal condition was a pip content of approximately 51 wt% in the hybrid film, with dimethyl sulfoxide (DMSO) as the solvent. SWCNTs and pip were dispersed and hybridized in situ in the solvent for 0.5 h, followed by vacuum filtration to produce a self-supporting film. The SWCNT/pip hybrid exhibited excellent *n*-type thermoelectric performance at room temperature, with a Seebeck coefficient of −116.7 μV/K, a power factor of 291.0 μW/(m·K^2^), and a *ZT* of 0.029 (Figure 8b,c). As shown in Figure 8d,e, the deep LUMO level of pip (−4.68 eV) prevents oxidation by oxygen and water; consequently, even after 220 days (~7.3 months) under atmospheric conditions without encapsulation, the material retained more than 87% of its initial electrical conductivity, demonstrating outstanding long-term atmospheric stability [77].

**Figure 9:**
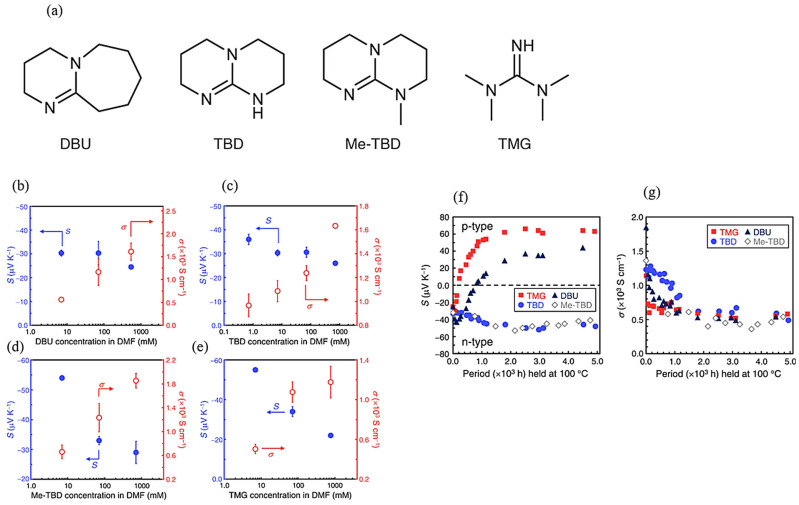
(**a**) Structural formulas of DBU, TBD, Me-TBD, and TMG. (**b**–**e**) Seebeck coefficient (*S*) and electrical conductivity (*σ*) as a function of dopant for DBU, TBD, Me-TBD, and TMG (*n* ≥ 3; error bars: SD). (**f**) Long-term stability of *S* and (**g**) *σ* in air at 373 K. Reprinted from [78] under the CC BY license.

Method 2: Exceptional heat resistance and atmospheric stability through doping with bicyclic organic superbases. Horike et al. developed a simple doping method using bicyclic organic superbases with strong electron-donating properties, realizing *n*-type SWCNTs that remain stable under both atmospheric and high-temperature conditions. Bicyclic guanidine- and amidine-based organic superbases (TBD, DBU, Me-TBD, TMG, etc.) were used as dopants for SWCNTs (eDIPS method). The structural formulas are shown in Figure 9a. The dopant concentration in solution was 71 mM (for TBD), with N,N-dimethylformamide (DMF) as the solvent. A pre-prepared *p*-type SWCNT self-supporting film (10–12 μm thick) was immersed in a DMF solution of the dopant at room temperature for 5 min (dip doping) and then vacuum-dried. As shown in Figure 9b–e, electron injection from the lone pairs of the organic bases reversed the Seebeck coefficient to −20 to −55 μV/K, with electrical conductivity ranging from 500 to 1800 S/cm; TBD and Me-TBD achieved peak power factors exceeding 100 μW/(m·K^2^). As shown in Figure 9f,g, when using TBD or Me-TBD, the Seebeck coefficient remained stable around −50 μV/K with *n*-type polarity even after continuous heating for 5000 h (~6 months) under harsh conditions of 373 K in air, demonstrating remarkable long-term thermal stability [78].

**Figure 10:**
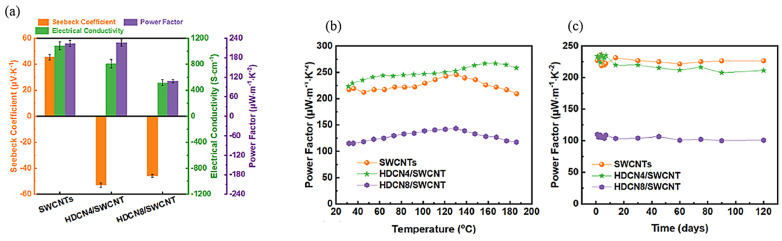
(**a**) Thermoelectric properties, (**b**) power factor variation with temperature, and (**c**) stability in air of SWCNT, HDCN4/SWCNT, and HDCN8/SWCNT composites. Reprinted with permission from Ref. [21]. Copyright 2024 American Chemical Society.

Method 3: Highly stable and robust flexible *n*-type SWCNT composite films using cross-linkable polymers with dynamic covalent networks. Xiao et al. reported *n*-type SWCNT composite films with exceptionally high atmospheric stability, utilizing a novel cross-linkable polymer (HDCN) featuring a hemi-aminal dynamic covalent network. Unmodified single-walled carbon nanotubes (SWCNTs) were used as the material; anhydrous ethanol was used as the solvent; and HDCN4 or HDCN8—cross-linkable polymers synthesized from diamine-terminated PEG and paraformaldehyde—were used as the *n*-type dopant and polymer matrix. SWCNTs and HDCN4 were mixed at an optimal mass ratio of 1:1 (20 mg:20 mg) and stirred for 6 h at 323 K to prepare a dispersion. The *n*-type film was fabricated as a self-supporting flexible film by vacuum filtration of this suspension onto a porous PVDF membrane, followed by overnight drying in a vacuum oven at 323 K. As a result, as shown in Figure 10a, we achieved an organic *n*-type thermoelectric material in which the HDCN4-doped *n*-type SWCNT film exhibited a high power factor (*P.F.*) of 225.9 [μW/(m·K^2^)] at room temperature. Regarding stability, as shown in Figure 10b, the ethylene glycol chains in the polymer strongly bind to defects in the SWCNTs, physically blocking oxygen; therefore, this *n*-type film exhibits excellent long-term atmospheric stability even without encapsulation, with a decrease in power factor of less than 10% over a period of more than 4 months (120 days). Furthermore, as shown in Figure 10c, it exhibited thermal stability (less than 3% weight loss) even at high temperatures up to 473 K [21].

**Figure 11:**
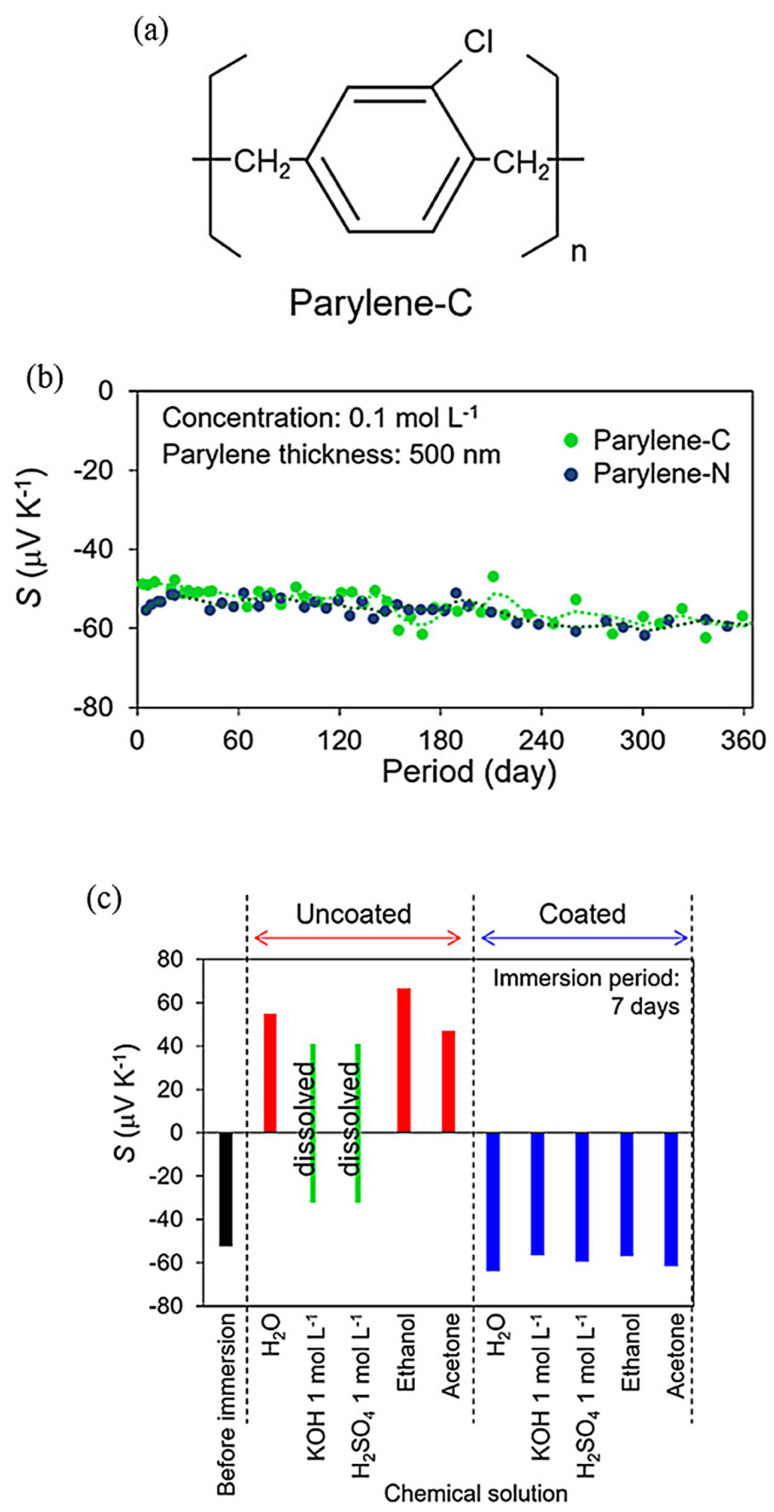
(**a**) Molecular structure of parylene-C. (**b**) Seebeck coefficient of parylene-C-coated *n*-type CNTs over 365 days. (**c**) Results of chemical durability tests in strong acids, strong alkalis, and alcohols over 7 days. Reprinted from Ref. [79] under the CC BY license.

Method 4: Exceptional long-term atmospheric and chemical stability through integration of supramolecular dopants and polymer protective layers. By separating the roles of electron injection (via *n*-type dopants) and protection (via a parylene coating), Suzuki et al. (2023) [79] created *n*-type SWCNTs with atmospheric stability exceeding one year and durability under harsh environmental conditions. A complex of benzo-18-crown-6-ether (B18C6) and potassium hydroxide (KOH) (dopant concentration 0.1 mol/L) was used as the dopant and parylene-C (~500 nm film thickness) as the protective layer. The molecular structure of parylene-C is shown in Figure 11a. Pure water (an aqueous solution of B18C6 and KOH) was used as the solvent. A film formed from the SWCNT dispersion by mask filtration was transferred to a polyimide substrate, immersed in the dopant aqueous solution to convert it into its *n*-type form, and then encapsulated by chemical vapor deposition (CVD) of parylene. Strong electron injection from the B18C6–KOH complex yielded an initial Seebeck coefficient of approximately −50 to −60 μV/K. As shown in Figure 11b, the robust parylene protective layer completely prevents the ingress of oxygen and moisture as well as dopant degradation due to physical friction; the Seebeck coefficient showed no degradation even after 365 days (over one year) in air. Furthermore, as shown in Figure 11c, the device maintained its *n*-type characteristics even after 7 days of immersion in strong acids, strong alkalis, and alcohols, demonstrating outstanding chemical durability and enabling thermoelectric power generation in strongly alkaline aqueous solutions—previously considered difficult [79].

In the development of atmospherically stable *n*-type SWCNT thermoelectric materials, organic approaches—including the use of organic small molecules with deep LUMO levels, doping with highly heat-resistant bicyclic organic superbases, the formation of defect-shielding barriers using polymers such as PEG, and the integration of supramolecular dopants with CVD polymer coatings—have proven highly effective. Through appropriate material selection, formulation ratios, solvents, and fabrication processes, both atmospheric and thermal stability lasting from several months to over a year can be achieved alongside high power output, bringing next-generation flexible energy-harvesting devices and photothermal sensors for harsh environments closer to practical application.

Additional approaches utilizing polymers and organic compounds are summarized in Table 3.

## 4. Flexibility of *n*-Type SWCNT Films

While metal-based inorganic materials such as bismuth telluride (Bi_2_Te_3_)—the mainstream thermoelectric conversion materials to date—exhibit excellent thermoelectric performance, they present several challenges: high rigidity and susceptibility to brittle fracture, toxicity to humans and the environment, and increased manufacturing costs due to complex processing.

In particular, when these materials are applied as self-powered sources for wearable devices and IoT sensors, they must conform seamlessly to curved surfaces or irregular heat sources such as the human body or piping. With highly rigid bulk materials, adhesion at the interface with the heat source decreases and thermal resistance increases, hindering efficient heat absorption and making it difficult to achieve a sufficient temperature difference (and thus power output). Furthermore, mechanical robustness capable of withstanding bending and tensile stresses—caused by body movements in wearable applications and by vibrations in IoT installation environments—is essential. The introduction of flexibility into thermoelectric devices is therefore considered one of the most critical challenges for societal implementation [82,85,100,110,111,112].

Once flexibility is achieved, conformal (shape-adaptive) thermoelectric generator (TEG) modules that fit seamlessly onto any curved surface can be constructed. This is expected to enable a wide range of next-generation applications, including the fully autonomous operation of wearable devices and multifunctional smart sensors [21,37,76].

This section highlights four representative examples of thermoelectric devices reported in recent years that achieve both extremely high flexibility (bending resistance, stretchability, self-healing, etc.) and excellent device performance.

Example 1: Fully Printed All-Carbon Thermoelectric Generator Using Cationic Surfactants and an Aqueous Process. Mytafides et al. reported a highly efficient, fully printed, all-carbon organic thermoelectric generator (OTEG) without metal junctions, utilizing an environmentally friendly aqueous process. They used unmodified single-walled carbon nanotubes (SWCNTs) as the material, deionized water as the solvent, and CTAB, a cationic surfactant, as the *n*-type dopant. By mixing SWCNTs and CTAB at an optimal mass ratio of 10:14 (mg/mg) and subjecting the mixture to chip-type ultrasonic treatment, the researchers prepared a high-quality aqueous ink with a viscosity suitable for printing (approximately 350 cP). The *n*-type film was fabricated by blade-coating this ink onto a flexible Kapton substrate and drying it at 363 K for 20 min.

**Figure 12:**
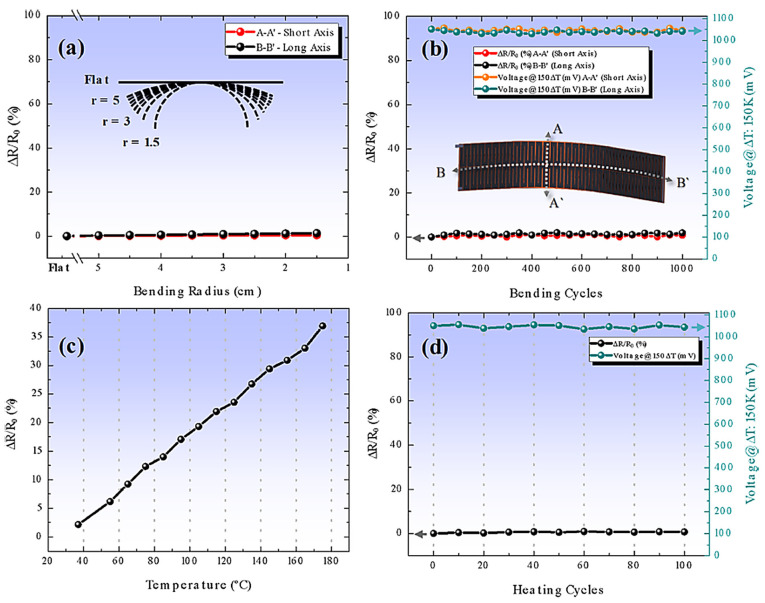
Evaluation results regarding the flexibility and reliability of the developed OTEG device. (**a**) Measurement results of the resistance change rate (Δ*R*/*R*_0_) of the OTEG under various bending radius conditions. (**b**) Changes in Δ*R*/*R*_0_ and output voltage as the number of bending cycles varies under a condition of Δ*T* = 423 K. (**c**) Behavior of Δ*R*/*R*_0_ as a function of temperature. (**d**) Stability (repeatability) of the OTEG’s Δ*R*/*R*_0_ and output voltage obtained after repeating 100 heating cycles at Δ*T* = 423 K. Reprinted with permission from Ref. [43]. Copyright 2021 American Chemical Society.

Because this module features an “all-carbon structure” without metal electrodes, it offers exceptional flexibility. As shown in Figure 12a–d, when the device was bent to a bending radius of 1.5 cm, the rate of change in internal resistance (Δ*R*/*R*_0_) was only 0.4% in the short-axis direction and 1.3% in the long-axis direction. Furthermore, even after undergoing 1000 consecutive bending cycles at a bending radius of 3 cm, the resistance change rates remained at 0.8% and 1.9%, respectively, demonstrating high fatigue resistance with a stable output voltage. Taking advantage of this flexibility, tests were also conducted using the device as a wearable power generator wrapped directly around a human arm. It stably generated 2.1 μW of power from a slight temperature difference (Δ*T* = 6 K) relative to body temperature and successfully lit an LED via a step-up converter [43].

**Figure 13:**
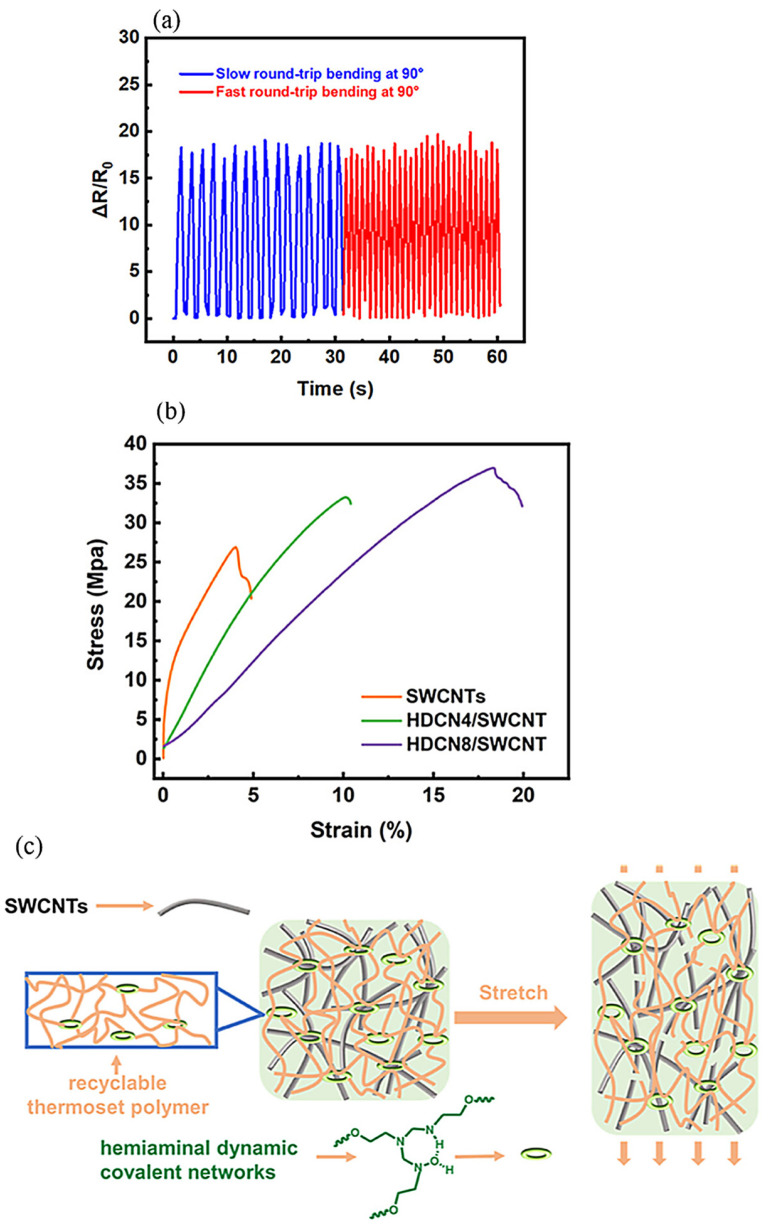
(**a**) Resistance response (Δ*R*/*R*_0_) of the HDCN4 composite self-powered sensor at a 180° bending angle. (**b**) Mechanical properties of untreated SWCNT, HDCN4/SWCNT, and HDCN8/SWCNT composites. (**c**) Schematic of the mechanical improvement achieved by adding a crosslinking polymer. In (**c**), the orange chains represent the recyclable thermoset polymer, the gray rods the SWCNTs, and the green loops the hemiaminal dynamic covalent crosslinks; the arrows indicate the stretching applied to the composite. Reprinted with permission from Ref. [21]. Copyright 2024 American Chemical Society.

Example 2: *n*-type SWCNT composite using dynamic covalent network polymers (Xiao et al.). The mass ratio of SWCNTs to a newly synthesized polymer (HDCN4 or HDCN8) varied from 1:1 to 1:5; the highest thermoelectric performance occurred at 1:1 (20 mg of polymer to 20 mg of SWCNTs). SWCNTs were ultrasonically dispersed in 20 mL of anhydrous ethanol for 6 h (300 W, 40 kHz), while the polymer was simultaneously dissolved in 10 mL of ethanol at 323 K. The two solutions were mixed and stirred for an additional 6 h at 323 K, then vacuum-filtered through a PVDF membrane (0.22 μm pore size) and dried overnight in a vacuum oven at 323 K. For evaluation, the device was worn on a finger as a self-powered sensor and tested at bending angles of 90° and 180°. As shown in Figure 13a, 65 consecutive sensing cycles under temperature differences (Δ*T*) of 10 K and 2.5 K confirmed stable voltage output without degradation. Even after 4 months in air without encapsulation, performance degradation was kept below 10%. Tensile strength was measured using a microprocessor-controlled universal testing machine (SANS-CMT4204) at a speed of 2 mm/min, with at least three film samples tested for each specimen. The initial non-linear region observed in the stress–strain curves reflects a toe region caused by slight slack and gradual seating of the film in the mechanical grips at the start of loading, rather than an intrinsic property of the material; the reported tensile strength and elongation-at-break values were therefore determined from the corrected, linear region of the curve after toe compensation. As shown in Figure 13b, at a SWCNT:polymer ratio of 1:1, HDCN8 (molecular weight 8000) yielded a tensile strength of 37.1 MPa and an elongation at break of 18%, owing to denser polymer chain entanglement, while HDCN4 (molecular weight 4000) yielded 33.2 MPa and 10%. The underlying mechanism is illustrated in Figure 13c [21].

Example 3: High-Performance Flexible Thermoelectric Generator Utilizing *n*-Type Doping via Oxidation of Adjacent Inorganic Sb_2_Te_3_. Kim et al. developed a flexible inorganic/carbon nanotube hybrid film with an extremely high power factor using a simple solution process that does not require a vacuum system. Bulk Sb_2_Te_3_ was dissolved in a mixed solvent of ethylenediamine and ethanethiol (volume ratio 4:1), and single-walled carbon nanotubes (SWCNTs) were added to this solution and uniformly dispersed using a ball mill to prepare a hybrid paste. By bar-coating this mixture onto a glass substrate and heat-drying it, Sb_2_Te_3_ recrystallized on the surface of the SWCNT bundles, forming a self-supporting hybrid film approximately 30 μm thick.

**Figure 14:**
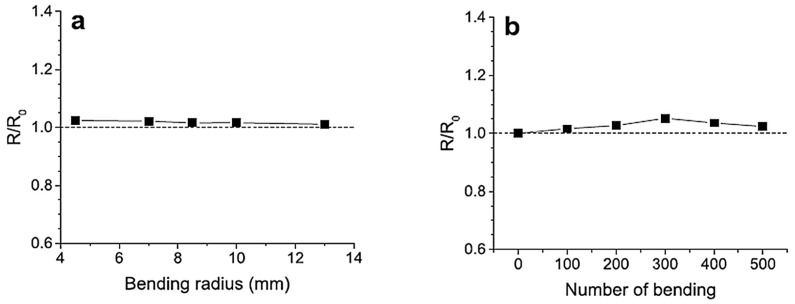
(**a**) Bending radius dependence and (**b**) bending cycle dependence of the relative resistance change in a hybrid film containing 37.5 wt% SWCNT. Reprinted with permission from Ref. [29]. Copyright 2020 American Chemical Society.

As shown in Figure 14a,b, the increase in electrical resistance when the film was bent to a sharp angle with a bending radius of 4.5 mm was limited to less than 2%. Furthermore, to demonstrate mechanical durability, the resistance increase remained below 2% even after 500 consecutive bending cycles at a bending radius of 4.5 mm. This indicates that the structure maintains high structural stability without the microscopic inorganic crystals on the surface peeling off or the network breaking due to bending stress [29].

Example 4: High-Performance Flexible Thermoelectric Generators via Oxygen Desorption and Molecular Doping of Directly Spun Carbon Nanotube Webs.

An et al. developed high-performance flexible thermoelectric generators (TEGs) by controlling the electronic structure of directly spun porous carbon nanotube (CNT) webs through heat treatment and molecular doping. An untreated, pure *p*-type CNT web was annealed for 10 h at 573 K in a nitrogen atmosphere to remove oxygen adsorbed on the surface, and then immersed for 8 h in a solution of benzyl viologen (BV), an *n*-type dopant (optimal concentration: 2 mg/mL). This preliminary oxygen-stripping process eliminated doping inhibition caused by oxygen, enabling highly effective *n*-type doping with BV.

**Figure 15:**
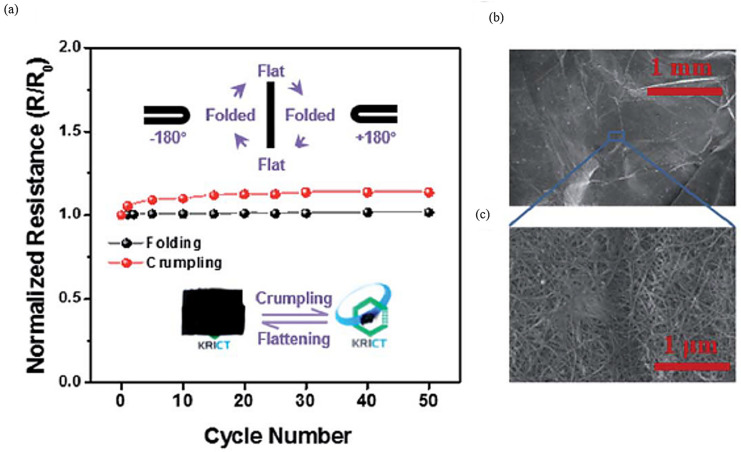
(**a**) Variation in resistance R as a function of the number of bending and crumpling cycles in a BV-doped a-CNT web (where *R*_0_ is the initial resistance before treatment). (**b**) Low-magnification SEM image of a BV-doped a-CNT web subjected to crumpling treatment, and (**c**) high-magnification SEM image of the same sample. Reprinted with permission from Ref. [45]. Copyright 2021 Royal Society of Chemistry.

The resulting TEG also exhibits excellent mechanical flexibility, reflecting the structural robustness of this porous CNT web. As shown in Figure 15a–c, even after repeating a folding test—in which the film was completely bent to +180° and −180°—50 times, the change in internal resistance was limited to less than 1.65%. To further demonstrate its mechanical strength, a “crumpling” test was performed, in which the film was intentionally crumpled into a ball and then flattened again. Even after 50 cycles of this extremely severe deformation, although wrinkles remained on the macroscopic film surface, the microscopic nanotube network was completely preserved without any breaks or collapse, demonstrating remarkable structural stability by limiting the change in resistance to approximately 13.6% [45].

Based on these results, flexible *n*-type thermoelectric materials have been successfully developed that simultaneously satisfy the requirement for mechanical toughness capable of withstanding severe deformation—an essential factor for practical application. These achievements will serve as a crucial foundational technology for realizing a next-generation sustainable electronics society, including rechargeable smartwatches, artificial skin, healthcare monitoring patches, and smart sensor networks for buildings.

## 5. Remaining Challenges and Prospects for Practical Application

This paper examines future challenges and prospects for the practical application of *n*-type SWCNT thermoelectric materials from five perspectives.

### 5.1. Large-Scale Manufacturing

One of the greatest barriers to commercialization is the transition from laboratory-scale methods to industrial-scale production. The bulk molding processes used for conventional inorganic thermoelectric materials—which involve high temperatures and high pressures—impose significant burdens in terms of both time and cost. To achieve commercialization, it is essential to develop large-scale, continuous solution-based and printing processes, such as screen printing, blade coating, and roll-to-roll (R2R) methods. Furthermore, developing methods to prevent SWCNT agglomeration and prepare high-quality ink in large quantities is a critical challenge. In this regard, it is essential to adopt continuous flow processing methods—such as high-speed laminar dispersion, which enables large-scale processing while maintaining crystallinity—instead of ultrasonic treatment, which can cause structural damage to SWCNT due to strong shear forces. Combining these approaches is expected to resolve various challenges in industrial manufacturing processes [50,113,114].

### 5.2. Reproducibility

Minimizing variations in performance and stability across different studies is essential for building highly reliable devices. Even for *n*-type dopants reported as “stable in air” in one study, it is not uncommon for that stability to fail to be reproduced in another study. This likely reflects the fact that the stability of doping depends not only on the molecular structure of the dopant but also strongly on the diameter of the SWCNTs used. Furthermore, when compositing with inorganic metal materials, particle size variations and aggregation at the interface are likely to occur, making the process extremely sensitive to manufacturing conditions. To improve reproducibility, it is necessary not only to rigorously screen the SWCNT raw materials but also to establish chemical processes for the stable synthesis of high-quality ink [67,115].

### 5.3. Device Integration/Assembly

Even if the performance of individual materials (such as *ZT* and *P.F.*) is high, the challenge remains of how to minimize losses that occur during the device fabrication process. Although SWCNTs themselves have extremely low resistance, when bonding them to metal electrodes such as copper or silver during device fabrication, non-ohmic contacts are prone to occur due to physical contact defects or energy level mismatches. As a result, the internal resistance of the entire module remains significantly higher than the theoretical value. Therefore, the development of an all-carbon TEG—in which the entire material system, including the electrode interfaces, is unified using carbon—is a critical challenge. Furthermore, to ensure the TEG functions properly, the performance balance between the *p*-type and *n*-type legs must be appropriately adjusted. In addition, there are structural challenges regarding how to adapt current TEG device designs for wearable devices—such as biosensors—that require flexibility and miniaturization. Additionally, to realize wearable applications that are worn directly on the human body to harvest body heat, efforts to reduce thermal contact resistance with the skin and demonstrate operation under real-world conditions remain insufficient, necessitating comprehensive evaluation at the device level [50,98,116].

### 5.4. Long-Term Operational Stability in Real-World Environments

The greatest technical challenge is preventing the “reversion to *p*-type due to oxidation”—a phenomenon specific to *n*-type materials—and ensuring stable, long-term operation under harsh real-world conditions. Currently, in the field of organic thermoelectricity (OTE), no standard protocol (conditions such as temperature, humidity, and evaluation period) has been established for assessing stability. As a result, researchers report “stability” based on their own criteria, making objective comparisons between studies difficult.

Furthermore, the causes of degradation are not limited to atmospheric oxygen. It has been clarified that moisture in the atmosphere (humidity) causes deliquescence and hydrolysis of *n*-type dopants, and that physical stresses such as bending lead to the destruction of the dopant layer—both of which are factors that directly contribute to the loss of *n*-type characteristics.

Therefore, to ensure long-term stability under real-world conditions, it is essential to develop a hydrophobic and flexible protective layer that can withstand physical friction while blocking both oxygen and moisture. Parylene coatings and SIS elastomers are cited as promising candidates [68,81].

### 5.5. Cost Aspects

For commercialization, it is necessary to dramatically reduce the cost per unit of power generated.

High-purity semiconducting SWCNTs (s-SWCNTs), which exhibit the best thermoelectric performance, are extremely expensive to manufacture and isolate. For practical application, it is crucial to develop technologies that enhance performance by heavily N-doping inexpensive, unseparated mixed SWCNTs, or to adopt approaches that repair and utilize defects in inexpensive SWCNTs—which are mass-produced in factories but have high defect densities.

Furthermore, as an alternative to expensive and toxic inorganic thermoelectric alloys (such as Bi and Te), the key to achieving commercialization lies in reducing overall costs—including not only material costs but also labor and manufacturing equipment costs—by utilizing fully automated laser processing methods and the aforementioned printing technologies [43,117,118].

For the practical application of *n*-type SWCNT thermoelectric materials, simply improving the performance of the material itself is insufficient; the fundamental challenge lies in the fact that the various issues—manufacturing, reproducibility, device integration, long-term stability, and cost—are interlinked. The immaturity of technologies for mass-producing low-cost, high-quality materials directly leads to low reproducibility across studies and performance losses during device integration. Furthermore, the absence of standard evaluation criteria for verifying long-term stability in real-world environments makes it even more difficult to properly resolve these challenges. Ultimately, all of these challenges converge on a single point: cost. Therefore, the practical application of *n*-type SWCNT thermoelectric materials cannot be achieved through a single technological breakthrough, but only through an integrated approach that consistently optimizes everything from material development to process standardization, device design, and durability evaluation.

## 6. Conclusions

This review has outlined the latest advances aimed at improving the atmospheric stability and flexibility of *n*-type SWCNT-based thermoelectric materials—two major challenges that have hindered the practical use of these flexible, environmentally friendly materials as self-powered energy sources for next-generation IoT and wearable devices. Although conventional metal-based inorganic materials exhibit high thermoelectric performance, their practical application has been limited by high rigidity and brittleness, the need for complex fabrication processes, and the presence of toxic heavy metals. To overcome these limitations, advanced compositing approaches that combine flexible, conductive SWCNTs with various materials at the nanoscale have seen significant development.

First, regarding *n*-type conversion and atmospheric stability through advanced compositing: several groundbreaking strategies have been established to address the greatest weakness of *n*-type SWCNT thermoelectric materials—performance degradation (reversion to *p*-type) caused by oxidation and moisture adsorption in air. In organic and polymer-based approaches, the use of organic small molecules with deep LUMO levels, doping with highly heat-resistant bicyclic organic superbases, and the formation of protective films using polymers or parylene coatings have proven extremely effective. These approaches achieve both stable *n*-type conversion through strong electron injection and a physical barrier function that prevents oxygen adsorption, realizing extreme stability and high output for several months to over a year in atmospheric and harsh high-temperature environments. In the inorganic hybrid strategy, coating and compositing SWCNTs with inorganic nanocrystals such as Bi_2_Te_3_ and Cu_2_O synergistically harnesses the high Seebeck coefficient of inorganic semiconductors and the high electrical conductivity of SWCNTs. Furthermore, by strongly scattering phonons through discontinuous chemical bonds at interfaces and introduced defects, the thermal conductivity—a key challenge for SWCNTs—is dramatically reduced while electrical conductivity is maintained. The inorganic layer also physically blocks contact with oxygen, effectively preventing re-*p*-type conversion even during long-term atmospheric exposure.

Methods using cationic or gemini surfactants serve dual roles, acting as dopants through electron donation and providing a physical oxygen barrier (bulk encapsulation) via high-density, tight adsorption onto the SWCNT surface. This has enabled ultra-long-term atmospheric stability spanning years while employing an aqueous process with low environmental impact. Significant progress has also been made in environmentally friendly, highly biocompatible processes utilizing natural amino acids such as lysine.

Second, regarding the flexibility and mechanical toughness essential for practical application, mechanical robustness is underpinned by mechanisms such as strong intermolecular interactions between SWCNTs and the surrounding polymer or coating, together with the formation of dense, cross-linked network structures that distribute stress across the composite. To ensure the physical robustness required to withstand vibrations from body movements in wearable applications and from IoT installation environments, strong hydrogen-bond networks formed between elastomer main chains (such as water-based polyurethane) and amino acids, as well as cross-linkable dynamic covalent network polymers, have been introduced. As a result, *n*-type thermoelectric composite materials have been established that simultaneously satisfy practical-level flexibility and fatigue resistance—capable of withstanding thousands of bending cycles and high tensile stress. These achievements directly enable conformal thermoelectric power generation modules that adhere seamlessly to the curved surfaces of the human body and to irregular heat sources, allowing efficient heat absorption. As we have reviewed thus far, methods for improving the air stability of *n*-type SWCNTs can be broadly classified into three strategies: “organic and polymer-based,” “inorganic hybrid,” and “surfactant and bio-derived.” Below, we discuss the advantages and limitations of each approach.

Organic and polymer-based composite materials offer the best balance between performance and stability. In particular, systems utilizing organic small molecules with deep LUMO levels or cross-linked polymers (such as HDCN4) that form dense networks have demonstrated long-term atmospheric stability lasting from several months to over a year (approximately 120–370 days), even without encapsulation. Although the figure of merit remains at a medium-to-high level of several hundred μW/(m·K^2^), these materials surpass other methods in terms of flexibility—such as elongation at break and flexural resistance—and are therefore considered the most practical option for wearable applications.

Inorganic hybrid composite materials, by contrast, exhibit high power factors derived from the intrinsic performance of conventional inorganic thermoelectric materials such as Bi_2_Te_3_ and Cu_2_O, reflecting the synergistic combination of their high Seebeck coefficient with the electrical conductivity of SWCNTs. However, compared with organic and polymer-based systems, these materials are generally inferior in terms of flexibility and long-term atmospheric stability, owing to the intrinsic brittleness and defect sensitivity of the inorganic component.

Surfactant- and bio-derived composite materials excel in mass producibility through low-cost, environmentally friendly aqueous processes, and their use of natural or commercially available surfactants and amino acids substantially reduces environmental impact compared with organic-solvent-based systems. However, because the heat resistance of the constituent organic molecules is inherently limited, these materials are generally unsuitable for medium- to high-temperature applications, restricting their use primarily to room-temperature or near-room-temperature wearable devices.

**Figure 16:**
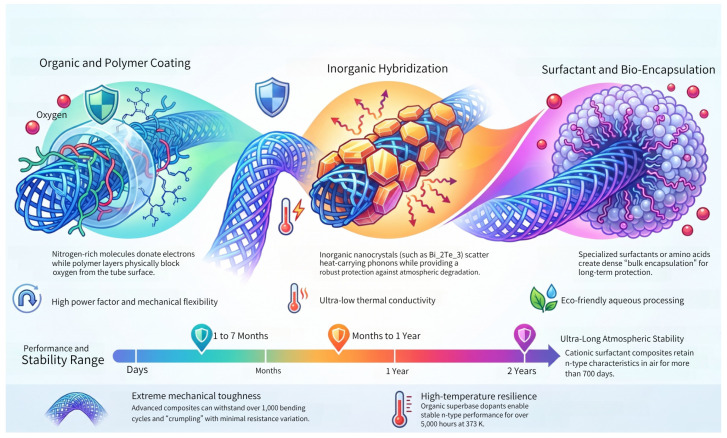
Powering the Future: Stabilizing Flexible *n*-Type Carbon Nanotubes. Conceptual overview of air-stabilization strategies for *n*-type SWCNTs, organized into three material systems—organic/polymer coating, inorganic hybridization, and surfactant/bio-encapsulation—each with its stabilization mechanism and characteristic advantage. The lower axis shows the atmospheric-stability duration achieved by each approach, and the bottom panels highlight the mechanical toughness and high-temperature resilience of the composites.

Figure 16 presents an overview of the various stabilization methods for *n*-type SWCNTs, organized according to material systems and stabilization mechanisms. In this figure, the methods are classified based on differences in material systems (organic/polymer-based, inorganic hybrid, and surfactant/biologically derived) and stabilization mechanisms, and the relationship between the corresponding thermoelectric performance (output factor, etc.) and stability in air is summarized.

This figure was created using Google’s NotebookLM (Pro version). To ensure accuracy and clarity and to avoid including unnecessary information, manual corrections and adjustments were made as needed using Canva (free plan). The author assumes full responsibility for the scientific accuracy of this figure and its compliance with copyright law.

Future prospects. Moving forward, the key to developing practical flexible thermoelectric modules will lie in integrating and selecting the optimization of composite materials, environmentally friendly solvent processes, and doping procedures according to the intended device applications. The highly stable, high-performance, and flexible thermoelectric materials created by these breakthroughs are expected to serve as a crucial foundational technology for driving the next-generation sustainable electronics society, including battery-free smartwatches, artificial skin, healthcare monitoring patches, and smart sensor networks for buildings.

## Figures and Tables

**Table 1:** Inorganic metal hybrid/SWCNT composites: materials, thermoelectric performance, and long-term stability.

SWCNT Type	Composite Material	Solvent	Fabrication Method	Seebeck Coeff. (μV/K)	Electrical Cond. (S/cm)	Power Factor (μW/(m·K^2^))	Long-Term Stability	Reference
SWCNT (floating catalyst CVD), ~300–373 K	Bi_2_Te_3_	Not specified	Magnetron sputtering (Ar, 0.8 Pa, 550 K, 120–720 s)	~−150	110–120	Not specified	No significant change in *S* or *σ* after 120 h at 473 K.	[54]
SWCNT (Nanjing XFNANO)	Bi_2_Te_3_	Deionized water	Hot press (80 MPa, 648 K)	−109.2 (340 K)	782.6 (300 K)	891.6 (340 K)	Not mentioned	[55]
SWCNT (XFS22)	Cu_2_O (PEG, SDBS)	Deionized water	Vacuum filtration	−17.6	278.3	5.89	210 days later: *σ* decreased from approximately 278 S/cm to approximately 240 S/cm (stable).	[30]
SG-CNT (Zeon)	Bi_2_Te_3_ (PVP)	EG (synthesis), MeOH/EtOH (film)	Solvothermal, drop casting, annealing (523 K, 1 h)	−130.0 (300 K)	81.8 (300 K)	138 (300 K)	After 500 bending cycles: *S* decreased by 11%.	[51]
TP8022 type (Shenzhen Nanotech Port)	SnSe (PVP)	EG, hydrazine hydrate	Solvothermal + vacuum filtration	−28.2 (RT)	740.1 (RT)	58.86 (RT)	Stable > 70 h under Ar; converts to *p*-type in air after 70 h.	[27]
SWCNT (Nanjing XFNANO)	Bi_2_Te_3_ (PVP)	EG	Solvothermal (in situ), vacuum filtration	−47.7 (386 K)	253.9 (386 K)	57.8 (386 K)	No degradation after 9 months in Ar; 90% *σ* retention after 300 bends.	[53]
Tuball SWCNT (OCSiAl)	Sb_2_Te_3_	Ethylenediamine/ethanediol (4:1)	Ball mill, bar coating, heat drying (423–473 K)	−150 (37.5 wt% SWCNT, RT)	~1100 (37.5 wt% SWCNT, RT)	2440 ± 267 (RT)	Not reported	[29]
SWCNT (Shenzhen Nanotech Port)	Bi_2_Te_3_ (cement-rebar structure, PVP)	Ethylenediamine, 1,2-ethanedithiol, EG	Drop casting, solvothermal, immersion, annealing (653 K)	−100.0 ± 1.7	517.2 ± 10.3	517.2 ± 26.2	*σ* and *S* remain nearly constant in air for 30 days.	[52]
SWCNT (floating catalyst CVD)	Bi_2_Te_3_ nanocrystals/CNT hybrid	Not specified	Magnetron sputtering, Ar plasma (100–800 s), annealing (630 K)	−145 to −147 (800 s)	75 (800 s)	Not specified	No change in *S* or *σ* after 120 h at 473 K.	[56]
SG-CNT (ZEONANO SG101)	Bi_2_Te_3_/DODMAC (cationic surfactant)	Deionized water, EG	Solvothermal, vacuum filtration, annealing (423 K)	−60 (after 2 h heat treatment)	15.3 (after 2 h heat treatment)	5.6	Cationic surfactant provides long-term air stability.	[57]

**Table 2:** Special materials, surfactants, and bio-derived SWCNT composites: thermoelectric performance and long-term stability.

SWCNT Type	Composite Material	Solvent	Fabrication Method	Seebeck Coeff. [μV/K]	Electrical Cond. [S/cm]	Power Factor [μW/(m·K^2^)]	Long-Term Stability	Reference
Tuball (OCSiAl), purity > 80%, d = 1.6 ± 0.4 nm	CTAB (cationic surfactant), SWCNT:CTAB = 10:14	Deionized water	Blade coating, 363 K drying	−41.8 (Δ*T* = 150 K)	1474 (Δ*T* = 150 K)	258	Without encapsulation in air: >100 days, change < 3%.	[43]
EC2.0 eDIPS-CNT (Meijo Nano Carbon), purity 90 wt%, d = 2.0 ± 0.7 nm	KOH + 18-Crown-6-ether	DMF	Immersion (impregnation) method	−33	2050	230	Stable at 373 K for >1 month in air.	[58]
SG101 (ZEONANO), purity > 99%, d = 3–5 nm	DODMAC/fluororesin	Ethanol	Heat treatment (423 K), fluororesin spray	−37	14.6	2.2	15 weeks in air.	[59]
SG101 (ZEONANO), purity > 99%, d = 3–5 nm	DODMAC	Deionized water	Drop casting on Japanese paper, heat treatment (423 K, Ar/H_2_)	−46	4.8	1.0	Ultra-long-term air stability (>2 years suggested).	[60]
SG101 (ZEONANO), purity > 99%, d = 3–5 nm	SDBS (anionic surfactant)	Deionized water	Drop casting, heat treatment (623 K, Ar/H_2_)	−45 (max)	35	5.3	*S* maintained ~−45 µV/K for 14 days in air; *p*-type conversion after 35 days.	[61]
EC1.5 eDIPS-CNT, purity > 90%, d = 1.5 nm	SDBS (anionic surfactant)	Deionized water	Drop casting, heat treatment (673 K, Ar/H_2_)	−44 (max)	386 (max)	34	*n*-type maintained for 14 days in air.	[61]
EC1.5 eDIPS-CNT, purity > 90%, d = 1.5 nm	Gemini surfactant (12-3-12)	Ultrapure water	Vacuum filtration	−37.8 (345 K)	1678 (345 K)	240 (345 K)	83% of initial *P.F*. retained after 120 days in air.	[37]
SWCNT (Nanjing XFNANO), purity 95 wt%, d = 1–2 nm	Lysine/water-based polyurethane (WPU)	NMP, water	Solution casting, vacuum drying	~−55.25	~350	94.3	90% *P.F*. retained after 72 h in air.	[39]
Tuball (OCSiAl), d = 1.6 nm	Ionic liquid [TBDH] [TFSI].	DMF	Electrochemical doping, vacuum drying	−30 (−0.25 V)	~1150	104	*n*-type maintained at 373 K for >1500 h (~2 months) in air.	[62]
SWCNT, purity > 85%, d < 3 nm (Shenzhen Nanotech Port)	CTAB (cationic surfactant)	DMF	Solution mixing, ultrasonic dispersion, vacuum filtration	−46.9 ± 2.3	843.5 ± 21.3	185.7 ± 8.5	Stable for >100 h in sealed plastic film.	[32]
SWCNT, purity > 85%, d < 3 nm (Shenzhen Nanotech Port)	Silver–ammonia complex/PEI	Anhydrous ethanol	Solution blending, vacuum filtration, NaBH_4_ reduction	−45.5	610.2	115.8	*n*-type maintained for 45 days in air (retention rate: 77.1%).	[63]
SWCNT, purity > 85%, d < 3 nm (Shenzhen Nanotech Port)	TBAB, SWCNT:TBAB = 10:5	DMF	Vacuum filtration	−55.4	1105	338.9	Seebeck coefficient remains at 90% even after 192 h in unsealed air.	[33]
SG101 (ZEONANO), purity > 99%, d = 3–5 nm	DODMAC + fluororesin coating on PPS mesh substrate	Ethanol	Dopant solution immersion, spray, heat treatment (423 K)	~−37	10.4	1.41	Negative Seebeck coefficient maintained for 15 weeks.	[59]
EC 1.5 (Meijo Nano Carbon)	Trinuclear metal macrocycle compound (Li^+^ receptor complex system)	Water	Drop casting on polyimide	−40.2 to −46.0 (330–390 K)	386–404 (330–390 K)	74.2 (max)	*n*-type conductivity maintained for 16 days at 373 K in air; Li^+^ hydration layer suppresses O_2_ penetration.	[64]
Tuball (OCSiAl)	ZnO nanowire (10 wt%) + PEI	Acetone/toluene (1:1)	Vacuum filtration	−23.5	777 ± 12	42.91 ± 2.7	Not mentioned	[65]
SG101 (ZEONANO), purity > 99%, d = 3–5 nm	DODMAC, SWCNT:DODMAC = 10:1 (mass ratio)	Deionized water	Vacuum filtration, heat treatment (423 K, Ar/H_2_)	−51	27–28	7	Stable in air for 280 days to >2 years.	[13]
SG101 (ZEONANO), purity > 99%, d = 3–5 nm	DODMAC	Deionized water	Vacuum filtration, heat treatment (423 K, Ar/H_2_)	−40 to −50	18–20	4.7	*n*-type characteristics maintained for >280 days in air.	[35]
SG101 (ZEONANO), purity > 99%, d = 3–5 nm	DODMAB	Deionized water	Vacuum filtration, heat treatment (423 K, Ar/H_2_)	−40 to −50	11–17	2	*n*-type characteristics maintained for >280 days in air.	[35]
SG101 (ZEONANO), purity > 99%, d = 3–5 nm	DODMAI	Deionized water	Vacuum filtration, heat treatment (423 K, Ar/H_2_)	−40 to −50	2.5–4	0.7	*n*-type characteristics maintained for >280 days in air.	[35]
SG101 (ZEONANO), purity > 99%, d = 3–5 nm	SC (sodium cholate)	Deionized water	Drop casting, heat treatment (523 K, Ar/H_2_)	−50 (max)	—	—	*n*-type retention lasted only ~6 days in air.	[61]
SG101 (ZEONANO), purity > 99%, d = 3–5 nm	DODMAC	Deionized water	Drop casting, heat treatment (423 K, Ar/H_2_)	−55	12	3.6	Stable *n*-type *S* (~−50 µV/K) for 665 days; *n*-type retained for 721 days.	[36]
SG101 (ZEONANO), purity > 99%, d = 3–5 nm	CPC	Deionized water	Drop casting, heat treatment (423 K, Ar/H_2_)	−50	—	—	*n*-type *S* (~−50 µV/K) maintained for 98 days; gradually decreasing; *p*-type after 120 days.	[36]
EC1.5P eDIPS (d = 1–3 nm)	Crown ether + KOH complex (1 M aqueous)	Pure water	Mask-filtration, immersion, 393 K drying	−31.4	2776	275	*n*-type maintained >180 days at 100 mM concentration (1 day at 25 mM).	[66]
SG101 (ZEONANO), purity > 99%, d = 3–5 nm	Crown ether + KOH complex (1 M aqueous)	Pure water	Mask-filtration, immersion, 393 K drying	−44.6	306	59	*n*-type maintained >180 days at 25 mM.	[66]
eDIPS EC2.0P (d = 2–4 nm)	Crown ether + KOH complex (1 M aqueous)	Pure water	Mask-filtration, immersion, 393 K drying	−29.1	2931	247	*n*-type maintained >180 days at 100 mM (20 days at 25 mM, 90 days at 50 mM).	[66]
Lamfil WPB-030 (CVD, d = 1.2–2 nm)	Crown ether + KOH complex (1 M aqueous)	Pure water	Mask-filtration, immersion, 393 K drying	−23.7	3694	217	*n*-type maintained >180 days at 100 mM.	[66]
SWCNT (EC2.0, d ≈ 2.5 nm)	Crown ether (DB18C6) + K^+^ complex, FePc	Ethanol	Bucky paper, K^+^–DB18C6 complex immersion, 383 K vacuum drying	−43	—	—	Stable in air.	[38]
SG101 (ZEONANO), purity > 99%, d = 3–5 nm	DODMAC on polyimide substrate	Ethanol	Vacuum filtration, heat treatment (473 K)	~−50	10–15	~3	Sufficiently flexible; applicable to curved surfaces.	[11]
CoMoCAT	K + benzo-18-crown-6-ether complex (1:1 or 3:1)	DMSO, THF, naphthalene	Vacuum filtration, 353 K annealing	~−60	~30	—	Thick films degrade in months; thin films degrade in minutes.	[67]
P2-SWNT	PEI/DODMAC	DMF	Drop cast, 473 K annealing	−29 (DODMAC 6%)	~400	~33	Adding DODMAC inhibits oxidation; equivalent to ~46 years at RT (Arrhenius extrapolation from 373 K testing).	[68]
HiPCO	K–crown ether complex or benzyl viologen (BV) + Al_2_O_3_ protective layer	Toluene, TFA	s-SWCNT extraction, ultrasonic spraying, polymer removal, Al_2_O_3_ coating	~−60	~1000	Stable in air for >300 h; performance decrease < 5%.	~300–400	[69]
Plasma-torch (PT) SWCNT	BV, PFPD (separation) + Al_2_O_3_ protective layer	Toluene, TFA, dry ice/methanol	s-SWCNT extraction, ultrasonic spraying, polymer removal, Al_2_O_3_ coating	−78.5	1190	730	*n*-type doping state confirmed after 3 days in air.	[69]
eDIPS SWCNT (Meijo Nano Carbon)	Alkali metal–crown ether complex (K:B18C6)	THF, DMSO	Stirring, vacuum filtration	−34.16 ± 3.1	—	—	*n*-type stability at 453 K more than doubled compared to additive-free.	[70]
EC 2.0 (Meijo Nano Carbon)	Methyltrioctylammonium chloride	Acetone, methanol	Filtration, vacuum drying, immersion doping	−59.4	626	221	Stable in air for >100 h.	[71]
EC 2.0 (Meijo Nano Carbon)	Tetraoctylammonium hydroxide	Acetone, methanol	Filtration, vacuum drying, immersion doping	−33.1	2076	227	Not specified.	[71]
EC 2.0 (Meijo Nano Carbon)	Tetraoctylammonium chloride	Acetone, methanol	Filtration, vacuum drying, immersion doping	−56.1	653	207	Not specified.	[71]

**Table 3:** Polymer and organic SWCNT composites: materials, thermoelectric performance, and long-term stability.

SWCNT Type	Composite Material	Solvent	Fabrication Method	Seebeck Coeff. (μV/K)	Electrical Cond. (S/cm)	Power Factor (μW/(m·K^2^))	Long-Term Stability	Reference
SWCNT (Nanjing XFNANO, d < 3 nm)	HDCN4 (crosslinkable polymer)	Anhydrous ethanol	Vacuum filtration	−52.9 (RT)	808.2 (RT)	225.9 (RT)	Stable unsealed in air for >4 months (>90% of properties retained); thermally stable up to 473 K.	[21]
SG-SWCNT (super-growth method)	Triphenylphosphine (tpp)-containing film	DMSO	Suction filtration (bucky paper), vacuum drying (353 K)	−272 (310 K)	~60 (310 K)	~25 (310 K)	Practically useful and highly air-stable in atmospheric environments.	[80]
SG-SWCNT (super-growth method)	dppp	DMSO	Suction filtration (bucky paper), vacuum drying (353 K)	−252 (310 K)	100 (max)	25	Practically useful and highly air-stable in atmospheric environments.	[80]
Meijo-SO (d ≈ 1.4 nm)	Cobaltocene-encapsulated SWCNT (CoCp_2_@SWCNT)	NMP	Filtration, 353 K vacuum drying	−41.8 (320 K)	432 (320 K)	75.4 (320 K)	TGA: thermally stable up to ~570 K in air and ~700 K in nitrogen.	[81]
SWCNT (Nanjing XFNANO, purity > 95%, d = 1–2 nm)	PEI/PMMA (2:8)	NMP	Vacuum filtration, vacuum drying (353 K)	−42.4 (RT)	1063.1	190.8	~72% of *P.F*. retained after 20 days in air; <5% mass loss up to 475 K.	[16]
SWCNT (Nanjing XFNANO, purity > 95%, d = 1–2 nm)	PEI/PHEMA	NMP	Vacuum filtration, vacuum drying (353 K)	−38.5	754.8	98.2	~72% of initial *P.F*. retained after 20 days.	[16]
SWCNT (XFNANO, d = 1–2 nm)	Acridine derivative (ADLA4)	DMSO	Vacuum filtration, 333 K vacuum drying	−60.7 ± 2.3 (RT)	529.6 ± 21.0 (RT)	195.2 ± 7.3 (RT)	*n*-type characteristics maintained after 380 h in air; stable up to ~581 K.	[19]
SWCNT (Shenzhen Nanoport)	PEI/TEG-C_60_ (modified fullerene)	Ethanol	Drop coating, 333 K drying	−42	923	162–167	Seebeck coefficient retained at 85% after 20 days in air at 393 K.	[44]
SWCNT (Nanjing XFNANO)	PEI/PVP composite	NMP, water	Drop casting, 353 K vacuum drying (2 h)	−28 ± 2 (300 K)	260 ± 40 (300 K)	21.2 ± 0.5 (300 K)	Seebeck coefficient unchanged after 115 h in air; thermally stable up to 441 K.	[82]
SWCNT (Meijo Nano Carbon EC2.0, purity > 90%)	1,1′-Bis(diphenylphosphino)ferrocene (dppf)	o-Dichlorobenzene	Filtration, 473 K heat treatment under vacuum	−38 (RT)	716 (RT)	110 (RT)	Stable for >18 months when sealed; *n*-type maintained after 5 days of air exposure.	[83]
SWCNT (Shenzhen Nanotech Port)	DETA, CaH_2_, PVP	Ethanol/water (1:4), anhydrous ethanol	Reduction treatment, vacuum filtration, 353 K vacuum drying	−41.0 ± 1.5 (RT)	165 ± 10	27.7 ± 2.3	Not reported	[17]
SWCNT (Shenzhen Nanotech Port)	PEI, Nafion	Ethanol	Suction filtration, 328 K vacuum drying	−40 (15 wt% PEI)	170 (15 wt% PEI)	25.5 (15 wt% PEI)	Nafion acts as an oxygen barrier; 15 wt% PEI sample becomes *p*-type after 12 days in air.	[84]
SWCNT (Chengdu Organic Chemicals, purity > 95%)	PEI	Distilled water	Filtration, 343 K vacuum drying, packaging	−34.7 (5 wt%)	~38 (5 wt% PEI)	Not reported	With tape sealing, *S* decreases from −35.9 to −24.4 µV/K over 7 days, then stabilizes near −24 µV/K; untreated sample becomes *p*-type after 9 days.	[85]
SWCNT (Tuball)	PC (2 wt%)/PVP (5 wt%)	None (melt mixing)	Melt mixing, hot press molding	−31.5	~0.05	~0.002	PC-based system loses *n*-type character after 18 months of storage.	[86]
SWCNT (Suzhou Institute of Nano-Tech)	PVP/PVDF (0.20:0.002)	EG, DMF	Wet chemical method, vacuum filtration, immersion, 353 K heat drying	~−55 (initial)	417	73.4	Coating provides 60 days of air stability and 30 days under high humidity (RH 60%).	[87]
SWCNT (Tuball)	Aramid nanofiber (ANF)	Deionized water	Suction filtration, 353 K heat drying	Not reported	790.0 (75 wt% SWCNT)	Not reported	TGA: thermally stable up to ~773 K in air.	[88]
SWCNT (P2-SWNT)	PEI	DMF	Drop cast, annealing (473 K, H_2_/Ar)	−28.4	581	~47	PEI begins to evaporate at 305 K; ~18% mass loss in accelerated degradation test (373 K, 2 h).	[68]
eDIPS SWCNT	PVA (dispersant: polyoxyethylene(4) lauryl ether)	Deionized water	Inkjet printing, 453 K annealing, drop casting	~−40	~0.7	~0.15	*n*-type maintained at a stable level for at least 3 weeks in air.	[89]
eDIPS SWCNT	PVAc (dispersant: polyoxyethylene(4) lauryl ether)	DMF, water	Inkjet printing, 453 K annealing, drop casting	~−40	~0.4	~0.08	*n*-type maintained at a stable level for at least 3 weeks in air.	[26]
eDIPS SWCNT	PVP (dispersant: polyoxyethylene(4) lauryl ether)	DMF, water	Inkjet printing, 453 K annealing, drop casting	~−25	~0.4	~0.02	Seebeck coefficient decreases over time in air but *n*-type maintained after 3 weeks.	[26]
eDIPS SWCNT	PVC (dispersant: polyoxyethylene(4) lauryl ether)	DMF, water	Inkjet printing, 453 K annealing, drop casting	~−7	~0.3	~0.01	Converts to *p*-type within one day in air.	[26]
SWCNT (Tuball)	SDBS + PEI (substrate: glass fiber; fixative: epoxy resin)	Water (with SDBS)	Blade coating on glass fiber, 363 K heat drying, epoxy injection and curing	−28.4 ± 4	1.561	0.12	Not reported	[90]
SWCNT (Tuball)	SDBS + PEI (substrate: glass fiber)	Water (with SDBS)	Blade coating on glass fiber, 363 K heat drying	−31 ± 1.5	1400 ± 4	134.5	Not reported	[90]
Tuball 75	Epoxy resin/amine curing agent (Ep/Tuball-1%)	Methanol	Vacuum injection into bucky paper (353 K)	−25.5 ± 0.2	2	0.002	Not reported	[91]
Tuball 75	Epoxy resin/amine curing agent (Ep/Tuball-35%)	Methanol	Vacuum injection into bucky paper (353 K)	−16.0 ± 2.1	103	0.264 (34.5 wt%)	Not reported	[92]
SWCNT (Tuball)	PC/THTDPCl (3 wt%)	None (melt mixing)	Melt mixing, compression molding (553 K)	−34.2	~0.07	~0.008	Not reported	[89]
SWCNT (Tuball)	PEEK/THTDPCl (3 wt%)	None (melt mixing)	Melt mixing, compression molding (553 K)	−37.1	~10	~0.01	Not reported	[89]
SWCNT (Nanjing XFNANO, d < 3 nm)	PEG/NaOH	Ethanol	Vacuum filtration, 333 K vacuum drying	−31.9	1708.5	173.8	Stable for 2 months in air (change < 8%).	[22]
SWCNT (Nanjing XFNANO, d < 3 nm)	PEG	Ethanol	Vacuum filtration, 333 K vacuum drying	−50.8	351.8	90.8	Not reported	[22]
SWCNT (XFNANO)	P(NDI-HTO)	Triethylamine	Drop coating, 393 K vacuum drying	~−35 (RT)	~590 (RT)	72.2 ± 1.5 (RT)	77% of output retained after 1 week in air; pyrolysis temperature > 473 K.	[92]
SWCNT (XFNANO)	P(NDI-TP)	Triethylamine	Drop coating, 393 K vacuum drying	~−35	460–480	~58.7	67% of output retained; pyrolysis temperature > 473 K.	[92]
SWCNT (Chengdu Organic Chemicals, TNSR grade, purity > 95%)	PEI (6 wt%)	Ethanol	Vacuum filtration, 334 K heat drying, surface coating	~−22	~300	~0.15	Not reported	[93]
SWCNT (Shenzhen Nanotech Port)	MoS_2_/SDBS	DMF	SDBS doping, vacuum filtration, 333 K heat drying	−32.8 ± 2.76	1165.7 ± 53.4	128.8 ± 7.4	Converts to *p*-type after 20 days of air exposure.	[94]
SWCNT (TCI Chemicals India)	Conjugated polymer (PNF222)	o-DCB/TEA	Printed onto polyimide substrate at room temperature	−53 ± 2.3	202 ± 19	64 ± 1.5	>200 h of operational testing under sealed packaging.	[95]
SWCNT (CNTRENE C100)	PMMA (substrate: Si or SiO_2_)	Deionized water	Dip coating, e-beam device fixation, vacuum annealing (425 K)	−14	800	Not reported	Re-exposure to air causes *n*-to-*p*-type conversion.	[96]
CoMoCAT	PEI, SDBS	Deionized water	Vacuum filtration, 353 K annealing	~−40	~15–50	Not reported	Not reported	[67]
SWCNT (Nanjing XFNANO, d = 1–2 nm)	(1-Pentyl)triphenylphosphonium fluoride (PTPF)	Ethanol	Vacuum filtration, 333 K drying	−37.8	2055	293.7	Stable for >100 cycles; atmospheric thermal stability above 353 K.	[97]
SWCNT (Nanjing XFNANO, d = 1–2 nm)	1-Methyl-3-octylimidazolium fluoride (MOIF)	Ethanol	Vacuum filtration, 333 K drying	−30.8	~1750	~150	Not reported	[97]
SWCNT (Nanjing XFNANO, d = 1–2 nm)	TBPF	Ethanol	Vacuum filtration, 333 K drying	−42.9	~1500	~250	Not reported	[97]
SWCNT (Nanjing XFNANO, d = 1–2 nm)	BPIF	Ethanol	Vacuum filtration, 333 K drying	−41.2	~1250	~200	Not reported	[97]
SWCNT (Nanjing XFNANO, d = 1–2 nm)	TBAF	Ethanol	Vacuum filtration, 333 K drying	−41.1	~1250	~220	Not reported	[97]
SWCNT (Nanjing XFNANO, d < 3 nm)	Liquid crystal mixture E7	Triethylamine	Drop cast, drying	−42.57 ± 2.93	530.27 ± 34.84	95.37 ± 24.71	Not reported	[98]
SWCNT (Jiangsu Xianfeng, d = 1–2 nm)	FcMA + PEG	NMP	Vacuum filtration, vacuum drying (358 K, 12 h)	−47.6 ± 2.3 (RT)	2299.8 ± 175.6 (RT)	600.6 ± 22.9 (RT)	The changes in *S* and *σ* remained below 14% after >900 h of air exposure.	[42]
SWCNT (CVD)	PEI (2.5 wt%)	Ethanol	Aerosol doping method, real-time spraying measurement (500 s)	~−50	2900–3000	708	Not reported	[99]
eDIPS-SWCNT (EC1.5)	TBD (71 mM)	DMF	Solution immersion, vacuum drying	−28	1210	>100	Maintained at 373 K for >6 months in air.	[78]
SWCNT (P2-SWNT)	PEI	DMF	Drop casting, annealing (473 K, H_2_/Ar)	~−29	582	47	Not reported	[68]
SWCNT (P2-SWNT)	PEI + DODMAC (~1:10)	DMF	Drop casting, annealing (473 K, H_2_/Ar)	~−34	200–220	~33	Accelerated degradation test at 373 K for 24 h corresponds to ~46 years of aging at room temperature (Arrhenius extrapolation).	[68]
SWCNT (Nanjing XFNANO, d = 1–2 nm)	p-Phenylenediamine (PDA) (1:1)	NMP	Vacuum filtration, 353 K drying	−51.6	2655.9	745.5 ± 22.5	*S* nearly constant for 240 h (10 days) in air; 70% of initial value retained after 720 h (~1 month).	[40]
SWCNT (Nanjing XFNANO, d = 1–2 nm)	TAPA (1:1)	NMP	Vacuum filtration, 353 K drying	~−53	~1800	~520	Not reported	[40]
SWCNT (Nanjing XFNANO, d = 1–2 nm)	TAPPD (1:1)	NMP	Vacuum filtration, 353 K drying	~−52	~1500	~400	Not reported	[40]
SWCNT (Nanjing XFNANO, d = 1–2 nm)	BTA (1:1)	NMP	Vacuum filtration, 353 K drying	~−30	~2800	~280	Not reported	[40]
SWCNT (Nanjing XFNANO, d = 1–2 nm)	BTEA (1:1)	NMP	Vacuum filtration, 353 K drying	~−53	~1000	~280	Not reported	[40]
SG-SWCNT (super-growth method, Zeon)	B18C6 + KOH mixed solution; parylene-C coating	Water	Filtration, transfer, immersion, CVD parylene coating	−50	Not reported	Not reported	Stable in air for >365 days (>1 year).	[79]
SWCNT (OCSiAl Tuball BATT H_2_O 0.4%)	CMC/PEI	Water, methanol	Additive and immersion doping, 403 K annealing	−36	860	95	70% of initial *P.F*. retained after 1 month in air.	[40]
CNT web	Benzyl viologen (BV)	Not reported	573 K heat treatment, immersion doping	−116	2228	3103	Very stable under nitrogen; stabilizes after 2 h of air exposure.	[45]
FCCVD SWCNT continuous network	PEI	Ethanol	Drop casting, 323 K drying	−64	3630	1500	Stable in air for 3 months (change < 5%).	[100]
SWCNT (P2 grade, Carbon Solutions)	PEDOT, TDAE	Deionized water	Spray application, 323 K heat treatment	−4300 (annealed)	~5.5 (annealed)	1050	Under polyimide encapsulation: *S* retained at 81% and *σ* at 92% for 17 days.	[101]
SWCNT (Suzhou Institute of Nano-Tech and Nano-Bionics, CAS)	PVP	Ethylene glycol	Ultrasonic dispersion, heating and stirring (483 K), vacuum filtration, 353 K drying	−54	~905	260	Not reported	[24]
SWCNT (Shenzhen Nanotech Port, 85 wt%)	NDINE (amino-substituted naphthalenediimide), SWCNT:NDINE = 10:10	DMSO	Vacuum filtration	−60.2	400	135 ± 14	After 100 h of air exposure: *σ* retained at 84.7%, *S* at 75.1%.	[28]
SWCNT (Shenzhen Nanotech Port, 85 wt%)	PDINE (amino-substituted perylenediimide), SWCNT:PDINE = 5:10	DMSO	Vacuum filtration	−52.4	500	112 ± 8	After 100 h of air exposure: *σ* retained at 83.5%, *S* at 70.0%; stable up to 503 K.	[28]
CVD single-wall CNT (SWCNT:DWCNT = 50:50)	PEI/DETA/NaBH_4_ (PEI:DETA = 67:33)	Deionized water	Vacuum filtration, immersion, reduction treatment	−86	52	38	Not reported	[102]
SWCNT (Nanjing XFNANO, 10 wt%)	PA66 (polyamide)	NMP	Solution casting	−56.0	112.5	35.4	80% of performance retained after 48 h in air.	[15]
SWCNT (Nanjing XFNANO, 10 wt%)	PA6 (polyamide)	NMP	Solution casting	−54.5	112.4	33.43	Not reported	[15]
SWCNT	CPE-PyrBIm4 (cationic conjugated polymer), SWCNT:CPE-PyrBIm4 = 1:1	Water/methanol (1:1)	Spin coating, drop cast	−41.6	~100	17.8	Stable at 373 K.	[103]
SWCNT	PFBT-PyrBIm4 (cationic conjugated polymer), SWCNT:polymer = 1:2	Water/methanol (1:1)	Spin coating, drop cast	−21 ± 3	~0.6	0.03	Not reported	[103]
SWCNT (XFNANO)	FcMA, SWCNT:FcMA = 1:7	NMP	Vacuum filtration, 353 K drying	−46.07 ± 0.50	2674.86 ± 136.32	567.54 ± 27.18	75% of Seebeck coefficient retained after 48 h in air.	[104]
SWCNT (XFNANO)	FeCp_2_ (ferrocene), SWCNT:dopant = 1:7	NMP	Vacuum filtration, 353 K drying	−45.52 ± 3.59	1346.22 ± 202.16	276.02 ± 26.11	75% of Seebeck coefficient retained after 48 h in air.	[104]
SWCNT (OCSiAl Asia Pacific, purity 93%)	pip (molecular content 51 wt%)	DMSO	Vacuum filtration	−116.7 ± 4.1	196.0 ± 9.3	291	87% of initial properties retained for >220 days in air.	[77]
SWCNT (OCSiAl Asia Pacific, purity 93%)	dm (molecular content 60 wt%)	DMSO	Vacuum filtration	−70.9	340.7 ± 39.5	Not reported	Reference material in the same study.	[77]
SWCNT (OCSiAl Asia Pacific, purity 93%)	py (molecular content 60 wt%)	DMSO	Vacuum filtration	−85.6	180.4 ± 4.4	Not reported	Reference material in the same study	[77]
SWCNT (OCSiAl Asia Pacific, purity 93%)	pz (molecular content 60 wt%)	DMSO	Vacuum filtration	−74.2	187.8 ± 6.4	Not reported	Reference material in the same study	[77]
SWCNT (XFNANO)	ADTAb, SWCNT:ADTAb = 1:1	DMSO	Vacuum filtration, 333 K drying, measured at 430 K	−64.6 ± 1.1	715.0 ± 7.2	289.4 ± 2.8	Not reported	[105]
SWCNT (XFNANO)	ADTAc, SWCNT:dopant = 1:1	DMSO	Vacuum filtration, 333 K drying	−7.5 ± 3.2	286.0 ± 25.1	1.6 ± 1.1	Not reported	[105]
SWCNT (XFNANO)	ADTAd, SWCNT:dopant = 1:1	DMSO	Vacuum filtration, 333 K drying	−27.5 ± 2.4	528.0 ± 44.5	39.8 ± 9.8	Not reported	[105]
SWCNT (XFNANO)	ADTAe, SWCNT:dopant = 1:1	DMSO	Vacuum filtration, 333 K drying	−46.8 ± 4.6	454.0 ± 23.4	96.1 ± 9.6	Not reported	[105]
SWCNT (XFNANO)	EtFc, SWCNT:dopant = 1:7	NMP	Vacuum filtration, 353 K drying	−45.52 ± 3.59	1346.22 ± 202.16	436.63 ± 14.78	*n*-type characteristics maintained after 48 h in air.	[106]
SWCNT (XFNANO)	PYB, SWCNT:PYB = 1:12	Ethanol/water (1:4)	Vacuum filtration, 353 K drying	−48.5 ± 1.4	~832.4	193.6	Superior to NaBH_4_ over 48 h in air.	[106]
SWCNT (XFNANO)	DEANB, SWCNT:dopant = 1:12	Ethanol	Vacuum filtration, 353 K drying	−36.3 ± 0.9	Not reported	58.6	Superior to NaBH_4_ over 48 h in air.	[106]
SWCNT (FCCVD method)	PEI (substrate: PET)	Ethanol	Drop casting, 323 K drying	−64 (1 wt% PEI)	3630	~1500	Stable in air for >3 months (change < 5%).	[100]
SWCNT (P2-SWCNT, Carbon Solutions)	SDBS, TDAE	Deionized water	Spray method, annealing (323 K), TDAE treatment	−1200	7.3	1050	Retained for 17 days under polyimide encapsulation (*S* at 81%, *σ* at 92%).	[101]
SWCNT (EC2.0)	TPM-CB (malachite green derivative)	Water (doping), o-DCB (film formation)	Immersion in pH 12 solution (17 h), 353 K vacuum drying	−59	497	172	Stable in air for 1 month (796 h).	[107]
SWCNT (P2-SWCNT, purity 90%, d = 4–5 nm)	PEI, SDBS	DMF, deionized water	Filtration, spray film formation, 333 K vacuum drying	−58	Not reported	Not reported	Maintained for at least several days in air.	[108]
SWCNT	PVDF, PEI	DMF	Drop casting	−46 ± 2	Not reported (1.1× improvement vs. prior)	Not reported	Not reported	[109]

## Data Availability

No new data were created or analyzed in this study. Data sharing is not applicable to this article.
